# COVID-19 Specific Immune Markers Revealed by Single Cell Phenotypic Profiling

**DOI:** 10.3390/biomedicines9121794

**Published:** 2021-11-29

**Authors:** Francesca Sansico, Mattia Miroballo, Daniele Salvatore Bianco, Francesco Tamiro, Mattia Colucci, Elisabetta De Santis, Giovanni Rossi, Jessica Rosati, Lazzaro Di Mauro, Giuseppe Miscio, Tommaso Mazza, Angelo Luigi Vescovi, Gianluigi Mazzoccoli, Vincenzo Giambra, on behalf of CSS-COVID 19 Group

**Affiliations:** 1 Institute for Stem Cell Biology, Regenerative Medicine and Innovative Therapies (ISBReMIT), Fondazione IRCCS “Casa Sollievo della Sofferenza”, 71013 San Giovanni Rotondo, Italy; f.tamiro@operapadrepio.it (F.T.); m.colucci@operapadrepio.it (M.C.); e.desantis@operapadrepio.it (E.D.S.); a.vescovi@operapadrepio.it (A.L.V.); 2 Bioinformatics Unit, Fondazione IRCCS Casa Sollievo della Sofferenza, 71013 San Giovanni Rotondo, Italy; d.bianco@css-mendel.it (D.S.B.); t.mazza@css-mendel.it (T.M.); 3 Department of Hematology and Stem Cell TraNSPlant Unit, Fondazione IRCCS “Casa Sollievo della Sofferenza”, 71013 San Giovanni Rotondo, Italy; giovannirossi.fr@gmail.com; 4 Cellular Reprogramming Unit, Fondazione I.R.C.C.S. Casa Sollievo della Sofferenza, Viale dei Cappuccini, 71013 San Giovanni Rotondo, Italy; j.rosati@css-mendel.it; 5 Clinical Laboratory Analysis and Transfusional Medicine, Fondazione IRCCS “Casa Sollievo della Sofferenza”, 71013 San Giovanni Rotondo, Italy; l.dimauro@operapadrepio.it (L.D.M.); g.miscio@operapadrepio.it (G.M.); 6 Department of Medical Sciences, Division of Internal Medicine and Chronobiology Laboratory, Fondazione IRCCS Casa Sollievo della Sofferenza, 71013 San Giovanni Rotondo, Italy

**Keywords:** COVID-19, SARS-CoV-2, immune cells, single-cell RNA sequencing, flow cytometry

## Abstract

COVID-19 is a viral infection, caused by the severe acute respiratory syndrome coronavirus 2 (SARS-CoV-2) and characterized by a complex inflammatory process and clinical immunophenotypes. Nowadays, several alterations of immune response within the respiratory tracts as well as at the level of the peripheral blood have been well documented. Nonetheless, their effects on COVID-19-related cell heterogeneity and disease progression are less defined. Here, we performed a single-cell RNA sequencing of about 400 transcripts relevant to immune cell function including surface markers, in mononuclear cells (PBMCs) from the peripheral blood of 50 subjects, infected with SARS-CoV-2 at the diagnosis and 27 healthy blood donors as control. We found that patients with COVID-19 exhibited an increase in COVID-specific surface markers in different subsets of immune cell composition. Interestingly, the expression of cell receptors, such as IFNGR1 and CXCR4, was reduced in response to the viral infection and associated with the inhibition of the related signaling pathways and immune functions. These results highlight novel immunoreceptors, selectively expressed in COVID-19 patients, which affect the immune functionality and are correlated with clinical outcomes.

## 1. Introduction

Coronavirus disease 2019 (COVID-19) is a viral infection, caused by the severe acute respiratory syndrome coronavirus 2 (SARS-CoV-2), and was declared a pandemic by the World Health Organization (WHO) [1]. In September 2021, the ongoing COVID-19 pandemic produced nearly 227,000,000 detected illness cases and 6,670,000 deaths worldwide. Following host cell binding, in the early phases of infection, virus particles remain confined to the upper and lower respiratory tracts, in particular in those cells expressing high numbers of the Angiotensin Converting Enzyme-2 (ACE-2) receptor [2], and exponentially replicates in order to spread to the inner parts of respiratory tracts, finally leading to a hyperinflammatory state [3,4,5].

Viral infection of host cells triggers the innate immune response, which explicates through the release of cytokines such as tumor necrosis factor-α (TNF α), granulocyte–macrophage colony-stimulating factor (GM-CSF), Interleukin-(IL-1), IL-6, and Interferon (IFN)-γ [6]. This immunochemical storm induces the early activation of dendritic cells, macrophages and NK-cells [7,8] and consequently, with a positive feedback mechanism, the later activation of a pathogen-specific adaptive response through T cells, B cells and antibodies [9]. The achievement of an adequate immune cell response as well as immune regulatory molecules and neutralizing antibodies could guarantee the elimination of the SARS-CoV-2 viral threat [10,11]. Conversely, the impairment of the adaptive immune machinery at this stage, coupled with immunochemical hyperactivation, can cause severe disease symptoms in COVID-19 patients [12,13,14]. A major risk of multiorgan failure has indeed been reported together with lung inflammation [15,16,17,18] and represents the main manifestation of life-threatening respiratory distress at the severe stage [18].

Understanding how the cells of the immune system in the peripheral blood change their proportions and gene expression in response to SARS-CoV-2, during the different stages of infection and disease progression until patient recovery, is of fundamental relevance, as it will provide insights into the mechanisms of immunity against SARS-CoV-2, and will assist clinicians in the evaluation of possible therapeutic strategies to prevent negative outcomes. In this scenario, the use of single-cell methodologies, such as single-cell RNA sequencing and multiparameter flow cytometry coupled with biochemical analysis of immunochemical molecules, would delineate an evolutionary profile of the individual immune cell population’s state and consequently the host response dynamics [19,20,21].

To address some of the major questions regarding the changes in the gene expression of immune cell surface markers and cellular pathways involved in the host response to SARS-CoV-2 infection, we performed a targeted RNA sequencing at the single-cell level (scRNA-Seq) of 399 genes commonly expressed in human immune cells on PBMCs from COVID-19 patients and a control group constituted by healthy blood donors. We also optimized a flow cytometry assay for the detection of different subsets of PBMCs to assess changes in lymphocyte subpopulations according to the COVID-19 patient’s pathological condition at diagnosis and after hospitalization. This combined approach of scRNA-Seq and flow cytometry has highlighted the immune cell populations, mostly altered in the course of SARS-Cov-2 infection and COVID-19-specific cell surface markers, highly differentially expressed in patients at the diagnosis compared to healthy subjects. Overall, the results depict the altered immunological landscape at the onset of COVID-19 disease. Additionally, these findings offer the rationale for the design of flow-based assays for the detection of COVID-19-specific immune cell subsets in the peripherical blood over the course of SARS-CoV-2 infection.

## 2. Materials and Methods

### 2.1. Human Samples and Count Blood Cells

After the approval from the Research Ethics Committee of the “Fondazione I.R.C.C.S. Casa Sollievo della Sofferenza” was obtained, blood samples were collected from 50 COVID-19 patients and 27 healthy blood donors, who were recruited as controls, and all tested negative for SARS-CoV-2 infection. Informed consent was obtained from all individuals according to the provisions of the Declaration of Helsinki. All participants were Caucasian, from the same geographic area, and 40% of them were female. The median age was 66 years. All blood samples from the 50 COVID-19 patients were collected at the onset of the infection and within 5 days from receiving a positive result from SARS-CoV-2 molecular testing. The demographic and clinical features of considered patients are reported in Table S1. The patients were subsequently subdivided by disease severity according to the WHO clinical progression scale [22] into mild (N = 40) and severe (N = 10). Specifically, patients with mild disease were symptomatic subjects who, at the peak of the disease, only received oxygen by mask or nasal prongs; instead, patients with severe disease required intubation and mechanical ventilation and/or died. Blood samples from 27 former COVID-19 patients who had mild (N = 24) and severe (N = 3) disease severity, were also collected within 2 days from receiving a completely negative result from SARS-CoV-2 molecular testing and reported as “Post COVID-19”. Complete blood counts and blood analyses were performed at the “Fondazione I.R.C.C.S. Casa Sollievo della Sofferenza” research hospital with the Sysmex-XT-4000i automated hematology analyzer, following standard procedures.

### 2.2. Isolation of Peripheral Blood Mononuclear Cells (PBMCs)

PBMCs were isolated from whole blood using Ficoll-Paque. Briefly, 6 mL of EDTA-anticoagulated blood was diluted with an equal volume of phosphate-buffered saline, pH 7.4 (PBS), containing 0.05 M ethylenediaminetetraacetic acid (EDTA; Invitrogen, Waltham, MA, USA). A total of 12 mL of diluted blood was layered over 24 mL of the Ficoll-Paque PLUS (GE Healthcare, Casoria, NA, Italy). Gradients were centrifuged at 400× *g* for 30 min at room temperature in a swinging-bucket rotor with no brake or acceleration. The cell interface was carefully removed by pipetting and washed with PBS-EDTA by centrifugation at 250× *g* for 10 min. PBMC pellets were suspended in ammonium chloride solution (Stemcell Technologies, Vancouver, BC, Canada) and incubated for 10 min at room temperature on a mixing platform in order to lyse contaminating red blood cells. Isolated PBMC were finally washed with PBS-EDTA and then cryopreserved in liquid nitrogen in fetal calf serum (FCS; Invitrogen, Waltham, MA, USA) containing 10% dimethyl sulfoxide (DMSO; Thermo Fisher Scientific, Waltham, MA, USA) and stored until required for downstream analyses.

### 2.3. Targeted RNA Sequencing at Single-Cell Level (ScRNA-Seq)

The expression of 399 transcripts relative to the immune cell system (Immune Response Panel HS, cat. 633750, BD, Biosciences, Franklin Lakes, NJ, USA) was performed at single-cell level on human PBMCs using the BD Rhapsody Single-Cell Analysis System platform (BD, Biosciences, Franklin Lakes, NJ, USA). Specifically, human mononuclear cells were stained with FITC-conjugated antibody against CD45 (cat. 555482, BD, Biosciences, Franklin Lakes, NJ, USA) and DAPI (cat. D9542, Calbiochem, Sigma, Merck KGaA, Darmstadt, Germany) for the exclusion of dead cells. Subsequently the CD45+DAPI- cell fraction was isolated by FACS sorting through MoFlo Astrios cell sorter (Beckman Coulter, Brea, CA, USA). Subsequently, viabilities and concentrations of purified cells were determined with the BD Rhapsody Scanner system after staining with viability dyes, Calcein AM (1:200 dilution; cat. #C1430, ThermoFisher, Waltham, MA, USA) and DRAQ7^TM^ (1:200 dilution; cat. #564904, BD Biosciences, Franklin Lakes, NJ, USA), and incubation for 5 min at 37 °C. Cells were counted using the Improved Neubauer Hemocytometer (INCYTO, Chungnam-do, Korea). Afterward, 100,000 CD45 + alive cells, FACS-sorted and derived from 25 COVID-19 patients, were pooled equally in 650ml cold BD Sample Buffer in order to generate two independent pools for a total of 50 patients. A BD Rhapsody cartridge was loaded with 10,000 pooled cells derived from each pool for single-cell separation for a total of two cartridges. A third BD Rhapsody cartridge was also loaded with 10,000 pooled cells derived from 27 healthy blood donors as a control. Single cells were isolated using Single-Cell Capture and cDNA Synthesis with the BD Rhapsody Express Single-Cell Analysis System according to the manufacturer’s recommendations (BD Biosciences, Franklin Lakes, NJ, USA). The 399 targeted transcripts of the BD Rhapsody immune response panel were amplified with the BD Rhapsody targeted amplification kit (cat. 633,774, BD, Biosciences, Franklin Lakes, NJ, USA) following the manufacturer’s instructions. Unwanted PCR products and other small molecules were excluded performing a side cleanup using the AMPure XP Beckman magnetic beads (cat. #A63880, Beckman Coulter, Brea, CA, USA). DNA quantity and quality control were performed using the Qubit^TM^ dsDNA HS Assay Kit (cat. # Q32851, ThermoFisher Scientific, Waltham, MA, USA) and the electrophoresis system Agilent 2200 TapeStation, cartridge (cat. #5067–5584, Agilent, Santa Clara, CA, USA). Three independent sequencings (one for each pool of patients plus controls) were performed in paired-end mode on the NextSeq 500 System (Illumina, San Diego, CA, USA) with the NextSeq 500/550 High Output Kit v2.5 (150 Cycles) chemistry to reach a depth of 12,000 reads per cell on average. Cells derived from the two pools of 25 patients and the pool of 27 healthy donors were only identified based on the unique Illumina indexes associated with the DNA library generated from each of the three cartridges.

### 2.4. ScRNA-Seq Data Analysis

Sequencing data were processed on the Seven Bridges Platform (https://www.sevenbridges.com/, accessed on 25 November 2021) for sample demultiplexing and the generation of expression sparse matrixes. UMI counting was carried out, including sequencing reads correction, recursive substation error correction (RSEC) and distribution-based error correction (DBEC). Accepted reads were aligned to the reference genome (GRCh38) for identification and quantification and subsequently the count genes matrix was generated. Only cells with mitochondrial read rate ≤ 30%, detectable genes ≥ 200 and genes expressed in 10 or more cells were considered to have passed the QC and further evaluated. Highly dimensional scRNA-Seq data were analyzed using the SeqGeq software (FlowJo) for visualization, clustering by Phenograph algorithm [23] and differential expression analysis. The workflow based on the SCANPY software was also applied [24,25]. The count matrices of various single-cell experiments were read using the Pandas Python library and cells from different experiments were concatenated in a single data frame, keeping track of the sample group origin (COVID or control), the counts for different genes and cell type. Batch effects across different datasets were correct by mutual nearest neighbors (MNN) algorithm through R-based default functions [26] (Figure S1). SCANPY software [24] was also employed to make a first data check and trim out outlier cells and genes on the basis of preliminary statistics results in order to increase the quality of the dataset: cells expressing less than 20 or more than 100 different genes were removed, as well as cells having less than 100 or more than 1000 counts. Subsequently, genes expressed in less than 10 cells were removed from the global list of genes. Finally, a further filter was applied to remove cells expressing less than 15 genes among the selected receptor genes. At the end, 5839 peripheral blood mononuclear cells (PBMCs) from the two groups of 25 COVID-19 patients and 7127 cells from the 27 healthy blood donors were normalized together with 340 genes using the mean of the total count per cell, converted to natural logarithms and scaled by the application of SCANPY default functions. Additional SCANPY package functions were also used to analyze data, searching for differentially expressed genes between COVID and control cells, and to graph the obtained results through dot plots and violin plots. The plots were refined using the Matplotlib library (https://ieeexplore.ieee.org/document/4160265, accessed on 25 November 2021). The Mann–Whitney U test (also known as Wilcoxon rank-sum test or Wilcoxon–Mann–Whitney test) was performed to find differentially expressed genes; the resulting *p*-values were corrected using the Benjamini–Hochberg method, in order to exclude possible false positives.

### 2.5. Cell Culture and Transfection

HEK-293T cell line was in vitro expanded in DMEM medium supplemented with 10% fetal bovine serum (FBS), 1mM sodium pyruvate, 2 mM L-glutamine, 100 units penicillin and 100 μg/mL streptomycin (ThermoFisher, Waltham, MA, USA). HEK-293T cells were transiently transfected with pcDNA3.1 vectors, encoding the receptor-binding domain (RBD) of SARS-CoV-2 protein S (spike) fused with sfGFP fluorescent marker (S-RBD #141184, Addgene, Watertown, MA, USA) or structural (E #141185; M #141186; N #141191; Addgene, Watertown, MA, USA) and non-structural (NSP1 #141367; NSP2 #141368; NSP4 #141369; NSP5 #141370; NSP7 #141373; NSP8 #141374; NSP9 #141375; NSP10 #141376; NSP11 #141377; NSP12 #141378; NSP13 #141379; NSP14 #141380; NSP15 #141381; Addgene, Watertown, MA, USA) SARS-CoV-2 recombinant proteins with Strep-Tag II synthetic peptide. An empty vector, expressing only the sfGFP was also included as a control. Cell transfection was achieved using Polyethylenimine Linear, MW25000, Transfection Grade (PEI 25K) (Polysciences, Inc., Warrington, PA, USA) as described previously [27]. After transfection, HEK-293T cells were in vitro expanded for 2 days and subsequently assessed for GFP and Strep-Tag II expression by flow cytometry using an anti-Strep-tag II primary antibody (cat. ab184224, AbCam, Cambridge, UK) and an anti-mouse IgG Alexa Fluor488-conjugated secondary antibody (cat. A-11001, ThermoFisher, Waltham, MA, USA).

### 2.6. Protein Extraction and Western Blot Assay

HEK-293T cells, transiently transfected with SARS-CoV-2 plasmids or with empty control were collected, washed in ice-cold phosphate-buffered saline and subsequently lysed in ice-cold 50 mM Tris-HCl (pH 7.4), 0.25% sodium deoxycholate, 1% Nonidet P-40, 150 mM sodium chloride, 1mM sodium orthovanadate, 1mM sodium fluoride, 2.5 mM sodium pyrophosphate, 1mM EDTA, 1mM phenylmethylsulphonyl fluoride, and protease inhibitor cocktail (cat #539,134, Calbiochem, Merck KGaA, Darmstadt, Germany). Whole cell lysates were quantified using the Pierce BCA protein assay kit (cat. 23,227, ThermoFisher, Waltham, MA, USA) and 25 μg of total proteins were incubated at 95 °C for 10 min, loaded on SDS-PAGE gels and then transferred to Hybond-ECL membranes (Amersham, Biosciences Corp, Amersham, UK). The membranes were blocked with 5% milk/0.3% TBS-Tween 20 at 4 °C for 1 h and then probed with primary antibodies against GFP (1:1,000 dilution; cat. ab290, AbCam, Cambridge, UK) or Strep-tag II (1:1000 dilution; cat. ab184224, AbCam, Cambridge, UK) and β-Actin (1:6000 dilution; cat. A1978, Sigma-Aldrich, Darmstadt, Germany). HRP-conjugated secondary antibodies (Cat. NEF812001EA, Perkin Elmer, Waltham, MA, USA) were used at 1:10,000 dilution. The chemiluminescent signal was detected with enhanced chemiluminescence (ECL) (cat.32,106, ThermoFisher, Waltham, MA, USA) and subsequently autoradiography.

### 2.7. Flow Cytometry Assays

To detect B cells interacting with SARS-CoV-2 structural and non-structural proteins, 50 μL of whole blood was incubated with 500 μL of ammonium chloride solution (Cat. #07850, Stemcell Technologies, Vancouver, BC, Canada). After red blood cell (RBC) lysis, cells were washed with DPBS and resuspended in 200 μL of DPBS with 35 μg of protein extracts, containing the recombinant SARS-CoV-2 proteins or sfGFP only as control. After 20 min of cell protein incubation at room temperature, cells were washed with DPBS and then stained with an anti-CD19 PE-conjugated antibody (cat. 12-0198-42, ThermoFisher, Waltham, MA, USA). The DRAQ7 fluorescent DNA dye (1:1,000 dilution; cat. D15106, ThermoFisher, Waltham, MA, USA) was also used to identify the fraction of total living cells. To perform the indicated multiparameter flow cytometry assay, PBMCs were isolated as previously described and then stained with the 13-color antibody panel reported in Table S2 in DPBS with BD Horizon Brilliant Stain Buffer (Becton Dickinson, Franklin Lakes, NJ, USA) for 20 min at room temperature. In this assay, the LIVE/DEAD™ Fixable Near-IR dead cell stain kit (cat. L34975, ThermoFisher, Waltham, MA, USA) was also included for identifying the fraction of total living cells. All flow cytometry analyses were performed on FACS Canto2 (Becton Dickinson, Franklin Lakes, NJ, USA) or MoFlo Astrios cell sorter (Beckman Coulter, Brea, CA, USA). The FlowJo (Becton Dickinson, Franklin Lakes, NJ, USA) and GraphPad-Prism 8.4.3 software were employed for the visualization and statistical analyses of data using the gating strategy reported in Table S3 and Figure S2 for the identification of different cell subpopulations of PBMCs with the considered panel.

### 2.8. Assessment of Cytokines and Chemokines Levels

The plasma samples of COVID-19 patients collected at diagnosis (N = 50) and after hospitalization (N = 27) as well as healthy blood donors (N = 27) were isolated from whole blood using Ficoll-Paque as previously described. The MACSPlex Cytokine 12 Kit (cat. 130-099-169, Miltenyi Biotec., Bergisch Gladbach, Germany) was employed for determining the concentrations of the following cytokines: GM-CSF, IFN-α, IFN-γ, IL-2, IL-4, IL-5, IL-6, IL-9, IL-10, IL-12, IL-17 and TNF-α. A total of 1 mL of each plasma sample was incubated overnight in the dark on a shaking platform (1400 rpm) at room temperature with 20 µL of MACSPlex Capture Beads and treated according to the manufacturer’s recommendations. After staining, the beads coated with capture antibodies against the reported soluble analytes were detected using FACS Canto2 (Becton Dickinson, Franklin Lakes, NJ, USA). The flow cytometry data were assessed using FlowJo (Becton Dickinson, Franklin Lakes, NJ, USA) and GraphPad-Prism 8.4.3 software for the visualization and statistical analysis of quantitative data.

## 3. Results

### 3.1. Immunological Alterations in Peripheral Blood of COVID-19 Patients

**Figure 1:**
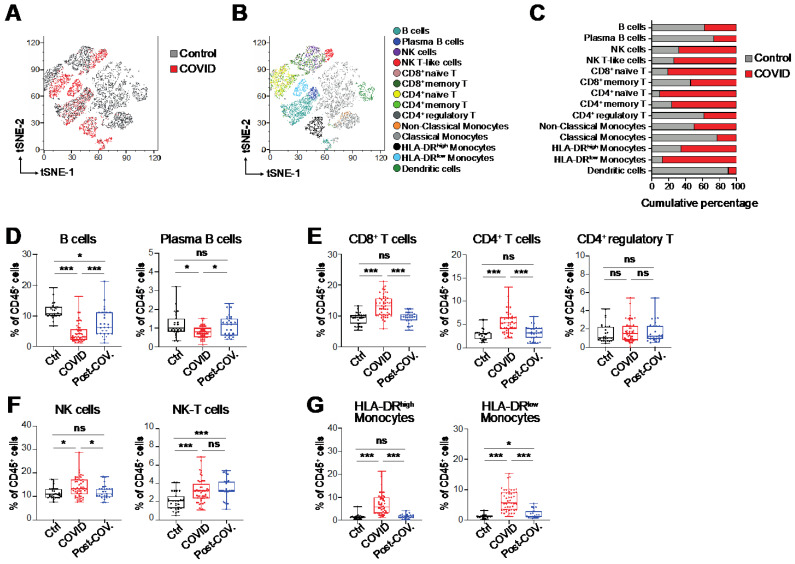
Distribution of peripheral blood mononuclear cells (PBMCs) from COVID-19 patients and control subjects by scRNA-Seq and flow cytometry profiles. (**A**) tSNE visualization based on the single-cell RNA sequencing (scRNA-Seq) profile from two groups of subjects, COVID-19 patients (N = 50) and healthy donors (N = 27) as control. A total of 5839 cells from the COVID-19 group and 7,127 cells from the control were considered for concatenation and t-SNE dimensional reduction using standard parameters (perplexity = 30, theta = 0.5). In the graph, each dot represents a single cell. Dots are colored by sample, red for cells from COVID-19 patients and grey for control. (**B**) tSNE plots as in (**A**) but with cells colored according to their assigned PhenoGraph cluster. Immune cell subsets were manually annotated based on the expression of gene markers as references according to the PanglaoDB web server [32]. (**C**) The graphical representation of cell percentage in the highlighted cell populations according to each sample group after down-sampling and normalization of cell numbers. (**D**–**G**) Distribution of PBMCs from COVID-19 patients at diagnosis (“COVID”, N = 50) and after the first negative molecular test for SARS-CoV-2 (“Post-COV”, N = 27) and healthy donor (“Ctrl”, N = 27) groups by multiparameter flow cytometry. PBMCs from each individual sample were labeled after red cell lysis with a panel of 12 fluorophore-conjugated antibodies against lineage-specific cell surface markers in order to identify the B, T, NK and monocyte cell subsets. Flow cytometry data are represented in distinct box plots for B cells and plasma B cells (**D**); CD8 + T cells, CD4 + T cells and CD4+ regulatory T cells (**E**); NK and NK-T cells (**F**); HLA-DR^high^ Monocytes and HLA-DR^low^ Monocytes (**G**). Welch’s t-test statistical analysis was performed on the percentage values of the different fractions of CD45+ cells. ns, not significant; *, *p* < 0.05; ***, *p* < 0.001 (two-tailed unpaired Welch’s t-test).

In order to highlight the immunological landscape and main changes in the gene expression of immune cell surface markers in COVID-19 patients, whole blood samples from 50 COVID-19 patients at diagnosis, as well as from 27 healthy blood donors as control, were collected at a large medical center in Southern Italy (Table S1) and analyzed by a targeted RNA sequencing system at the single-cell level (scRNA-Seq) (BD Rhapsody), which detects the expression of 399 transcripts relative to the immune cells system (Immune Response Panel HS, BD, Biosciences). In total, we analyzed 5839 peripheral blood mononuclear cells (PBMCs) from the group of COVID-19 patients and 7127 cells from the control. Initially, t-SNE dimensional reduction [28] was performed for the visualization of cell subsets in two dimensional plots using standard parameters (perplexity = 30, theta = 0.5). In this assay, the samples were divided in two main groups depending on whether they originated from COVID-19 patients (COVID) or healthy donors (control) (Figure 1A). We initially found increased proportions of leukocytes and monocytes in COVID-19 patients in line with previous reports [19,29]. Of interest, we observed a reduction in total and plasma B cells, and an expansion of CD8+ and CD4+ naïve T cells, natural killer (NK) and NK-T cell subsets, and HLA-DR^high^ and HLA-DR^low^ monocytes (Figure 1B,C). To validate these observations, we designed a multiparameter flow cytometry panel, including fluorophore-conjugated antibodies against surface lineage markers for the detection of discrete subsets of B, T and NK cell subsets and HLA-DR^high^ and HLA-DR^low^ monocytes (Tables S2 and S3). In order to assess the main immunophenotypic cellular changes before and after SARS-CoV-2 infection, we analyzed approximately 10^6 PBMCs from COVID-19 patients at diagnosis (“COVID”, N = 50) and after the first negative molecular test to SARS-CoV-2 (“Post-COV”, N = 27) and from healthy blood donors (“Ctrl”, N = 27) by multiparameter flow cytometry. We found a statistically significant reduction in total B and plasma B cells in COVID-19 patients at the diagnosis with respect to post-COVID and healthy donors (Figure 1D). On the contrary, the level of CD8+ and CD4+ T cells, NK and NK-T cell subsets and HLA-DR^high^ and HLA-DR^low^ monocytes within the CD14+CD16- classical compartment and total monocytes increased in response to the SARS-CoV-2 infection (Figure 1E–G and Figure S3). Of interest, a decrease in CD14+CD16- classical monocytes was detected in COVID-19 patients with respect to the other two groups, but no changes were noticed in the levels of CD14+CD16+ intermediate and CD14^dim^CD16+ non-classical monocytes between COVID-19 patients and healthy blood donors (Figure S3). Additionally, we observed an expansion of NK-T cells in former COVID-19 patients after hospitalization in the absence of SARS-CoV-2 virus as detected by molecular test. Interestingly, the level of HLA-DR^high^ and HLA-DR^low^ monocytes also increased according to disease gravity in patients with severe symptoms (Figure S4). Moreover, to evaluate any correlation with the proinflammatory innate immune response, we determined the concentration of soluble human cytokines and chemokines in the plasma and confirmed the increase in IL6, IFN-γ and IL4 in our cohort of COVID-19 patients with respect to other samples (Figure S5), in line with previous reports [30,31]. Finally, in order to highlight features that may have caused the increased morbidity in the considered COVID-19 patients, we performed a supervised regression analysis using all demographic, clinical and immunological data that were available for our cohort. Interestingly, we found that disease severity and mortality were statistically significantly correlated with the high levels of HLA-DR^high^ and HLA-DR^low^ monocytes, as well as with the patient’s age and the number of males (Figure S6). On the contrary, patient survival was primarily associated with the high level of total and plasma B cells (Figure S6). All these findings support the idea that a dysfunctional immune response enforces the disease pathology and correlates with the clinical outcome of COVID-19 patients.

### 3.2. Altered B Cell Signature and Activation States in COVID-19 Patients

**Figure 2:**
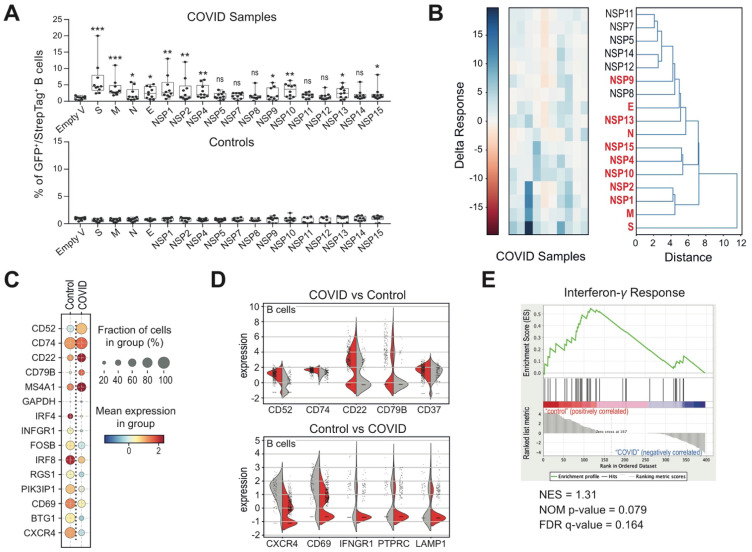
B cell signature and activation states in the peripheral blood of COVID-19 patients at the diagnosis. (**A**) Flow cytometry assessment of B cells, interacting with SARS-CoV-2 protein S (spike) fused with sfGFP fluorescent marker or structural (RBD-S, M, N and E) and non-structural (NSP1, NSP2, NSP4, NSP5, NSP7, NSP8, NSP9, NSP10, NSP11, NSP12, NSP13, NSP14, NSP15) SARS-CoV-2 recombinant proteins with Strep-Tag II synthetic peptide. The box plots represent GFP^+^ or Strep-Tag^+^ B cell fraction as a result of interactions between the GFP^+^/Strep-Tag^+^ recombinant SARS-CoV-2 proteins and PBMCs from 10 COVID-19 subjects at the top and 10 healthy donors as control at the bottom. Two-way ANOVA in combination with the Dunnett’s test statistical analysis was performed on the percentage values of B cells interacting with the different SARS-CoV-2 proteins. ns, not significant; *, *p* < 0.05; **, *p* < 0.01; ***, *p* < 0.001 (Two-way ANOVA with Dunnett’s test, comparing the indicated sample mean with the other values). *(***B**) On the left, the heatmap represents the level of interactions between COVID-19 patient’s B cells and each of the SARS-CoV-2 proteins. The graded color from red (negative) to blue (high positive) indicates the occurrence of the protein-interacting B cells. In the right panel, the hierarchical clustering of viral proteins is indicated on the basis of the different interactions with COVID-19 patient B cells. (**C**) Dot plot representation of genes, encoding cell surface proteins and highly differentially expressed among B cells from COVID-19 patients and healthy donors. The color and the size of each dot represent the average normalized expression levels and the percentage of cells expressing a given gene, respectively. On the vertical axis are listed the genes returned as significantly differentially expressed (*p* value < 0.01) while on the horizontal axis are displayed the cellular populations. (**D**) Violin plot representation of the five most differentially expressed receptor genes in B cell subsets from both cohorts of COVID (red) and control (grey) samples. (**E**) Gene set enrichment analysis (GSEA) of the “Interferon-γ response” hallmark gene set which was the most relevant hallmark gene set, identified as enriched in B cells from healthy donors in relation to COVID-19 patients. The horizontal bar in graded color from red (left) to blue (right) represents the GeneList ranked from high expression in the COVID subset indicated on the left to high expression in the control subset indicated on the right. Equivalent expression between the two subsets is reached at the red to blue border. The vertical black lines (‘bar code’) represent the projection onto the ranked GeneList of the individual genes of the GeneSet. The curve in green corresponds to the calculation of the enrichment score (ES). False discovery rate (FDR) q-values, normalized enrichment score (NES) and the corresponding nominal *p*-value (NOM *p*-value) are reported within the graph (Interferon-γ response GeneSet: NES = 1.31; NOM *p*-value = 0.079; FDR = 0.164).

B cells are well known as the key players of the adaptive humoral immune system and are responsible for the production of antigen-specific immunoglobulin directed against invasive pathogens [33]. In line with what has been already described in previous studies [34,35], we detected a decrease in B cells and plasma B cells in subjects with ongoing SARS-CoV-2 infection in contrast to control and post-COVID subjects. In particular, we observed a further decrease in B cells and plasma B cells in those patients displaying severe pathological conditions (Figure S4A). In order to investigate whether there had been a specialization of B cells towards the pathogen antigens despite the total population decrease, we planned a flow cytometry assessment of COVID subjects’ B cells interacting with SARS-CoV-2 structural and non-structural proteins (Figure S7). We found that statistically significant affinity was displayed by B cells towards the structural S, M, N and E and the non-structural NSP1, NSP2, NSP4, NSP9, NSP10, NSP13, and NSP15 viral proteins. The antigen-binding profile of uninfected healthy donors was also determined as control (Figure 2A), as well as the percentage of non-B cells, binding the various SARS-CoV-2 antigens, which was always lower than 1% in all considered samples (data not shown). Moreover, interacting viral proteins were grouped in clusters on the basis of each patient B cells’ response to the viral infection. In particular, besides the Spike protein, which represents the main viral protein towards which the action of B cells is concentrated, we identified three major clusters made up by M, NSP1 and NSP2; NSP10, NSP4 and NSP15; and NSP7 and NSP11 (Figure 2B). Nonetheless, no correlation was found with the disease severity and no statistically significant change between mild vs. severe patients was observed.

Subsequently, we analyzed the COVID-19-induced gene expression in all detected cell subsets, including B cells from COVID-19 patients in relation to the controls (Figure S8). Among the differentially expressed genes, we particularly focused on those genes encoding for surface proteins (Figure S9) with the intent to identify predictive cell markers of pathological condition or clinical outcome. Specifically, in the COVID-19 patient’s B cells, we detected a substantial increase in the expression of CD52, CD74, CD22 and CD79B and a decrease in CXCR4, CD69, INFGR1, PTPRC and LAMP1 transcripts with respect to control-derived B cells (Figure 2C,D). Of interest, the reduction in INFGR1 expression at a transcriptional level was in agreement with the gene enrichment of the interferon-γ signature pathways observed in B cells derived from healthy controls (Figure 2E), suggesting that the observed changes may have a direct effect on the signaling activity of B cells as well as on the virus-induced immune response.

### 3.3. Heterogeneous T and NK Cell Subsets in COVID-19 Patients

**Figure 3:**
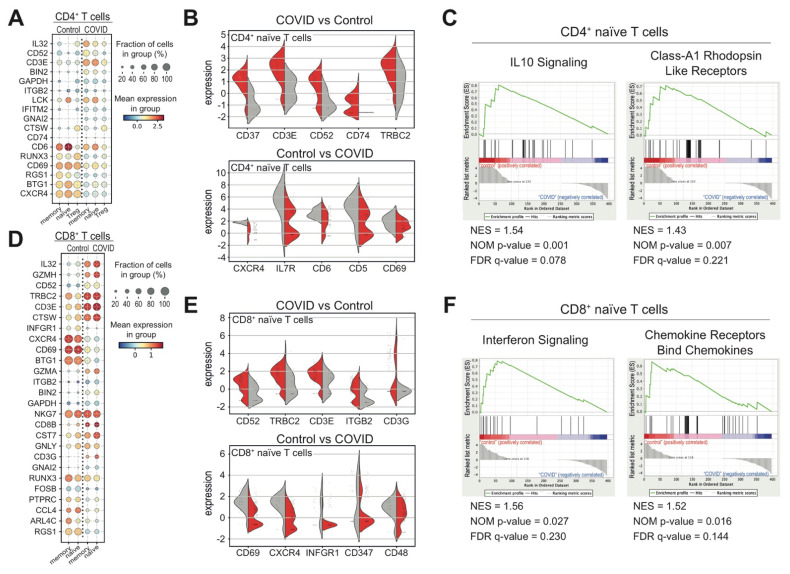
Transcriptional signature of CD4+ and CD8+ T cell subsets derived from COVID-19 patients at diagnosis. (**A**) Dot plot representation of genes, encoding cell surface proteins and significantly differentially expressed (*p* value < 0.01) among the indicated CD4+ cell subsets from COVID-19 patients and healthy donors. (**B**) Violin plot representation of the five most differentially expressed receptor genes in CD4^+^ naïve T cells from both cohorts of COVID (red) and control (grey) samples. (**C**) Gene set enrichment analysis (GSEA) of “IL10 signaling” and “Class-A1 Rhodopsin like receptors” reactome gene sets, which were the most relevant gene sets identified as enriched in CD4^+^ naïve T cells from healthy donors with respect to COVID-19 patients. False discovery rate (FDR) q-values, normalized enrichment score (NES) and the corresponding nominal *p*-value (NOM *p*-value) are reported within each graph (IL10 signalling GeneSet: NES = 1.54; NOM *p*-value = 0.001; FDR = 0.078; Class A1 Rhodopsin-like receptors GeneSet: NES = 1.43; NOM *p*-value = 0.007; FDR = 0.221). (**D**) Dot plot representation of genes, encoding cell surface proteins and significantly differentially expressed (p value < 0.01) among the indicated CD8+ cell subsets from COVID-19 patients and healthy donors. (**E**)Violin plot representation of the five most differentially expressed receptor genes in CD8^+^ naïve T cells from both cohorts of COVID (red) and control (grey) samples. (**F**) Gene set enrichment analysis (GSEA) of “Interferon signaling” and “chemokine receptors bind chemokines” reactome gene sets, which were the most relevant gene sets identified as enriched in CD8^+^ naïve T cells from healthy donors with respect to COVID-19 patients. False discovery rate (FDR) q-values, normalized enrichment score (NES) and the corresponding nominal *p*-value (NOM *p*-value) are reported within each graph (Interferon signalling GeneSet: NES = 1.56; NOM *p*-value = 0.027; FDR = 0.230. Chemokine Receptor Bind Chemokines GeneSet: NES = 1.52; NOM *p*-value = 0.016; FDR = 0.144).

T and NK cells form heterogenous populations playing relevant roles in the innate and virus-specific immune response [21,36]. In our clustering analysis, T cells were initially subdivided into CD4^+^ naïve, CD4^+^ memory, CD4^+^ regulatory, CD8^+^ naïve and CD8^+^ memory cell types based on the expression of canonical markers. In our previous observations, we found a statistically significant increase in CD4^+^ or CD8^+^ naïve T cells in COVID-19 patients with respect to other sample cohorts (Figure 1E). In order to identify a COVID-specific immunophenotypic transcriptional signature in naïve T cells, we searched for differentially expressed genes, encoding cell surface proteins, between COVID-19 and control samples (Figure 3A,D). Specifically, we found an increased expression of CD37, CD3E, CD52, CD64 and TRCB2 markers and a reduction in CXCR4, IL7R, CD6, CD5 and CD69 markers in COVID-derived CD4^+^ naïve T cells with respect to control cells (Figure 3B). Of interest, the low expression of CXCR4 as well as the class-A1 Rhodopsin-like receptors in COVID-derived CD4^+^ naïve T cells resulted in a reduced responsiveness of the IL-10 signaling pathway (Figure 3C), which may contribute to immunodeficiency in patients [37]. Instead, in COVID-derived CD8^+^ naïve T cells, we detected an increased expression of CD53, TRBC2, CD3E, ITGB2 and CD3G markers and a reduction in CD69, CXCR4, IFNGR1, CD347 and CD48 markers with respect to control cells (Figure 3E). Remarkably, these transcriptional differences correlated also with a gene enrichment of the interferon signaling in CD8^+^ naïve T cells originated from healthy donors (Figure 3F).

**Figure 4:**
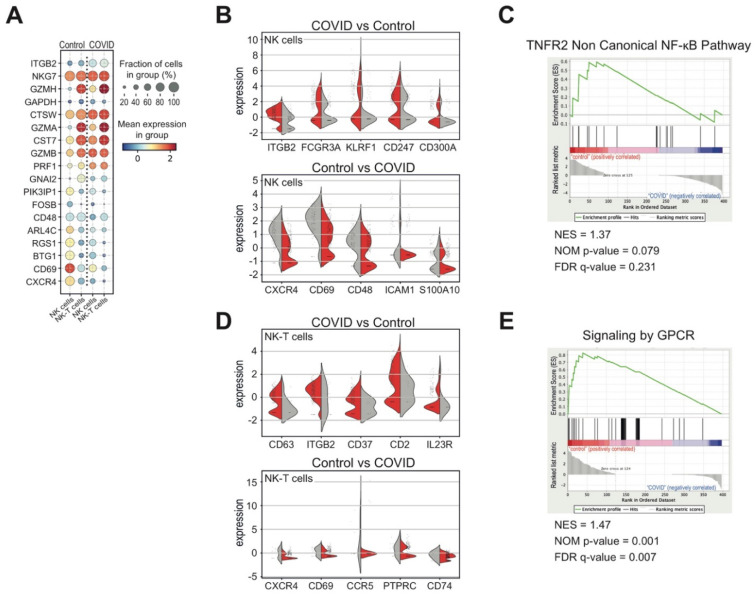
Transcriptional signature of NK and NK-T cell subsets derived from COVID-19 patients at diagnosis. (**A**) Dot plot representation of genes, encoding cell surface proteins and significantly differentially expressed (*p*-value < 0.01) among the NK and NK-T cell subsets from COVID-19 patients and healthy donors. (**B**) Violin plot representation of the five most differentially expressed receptor genes in NK cells from both cohorts of COVID (red) and control (grey) samples. (**C**) Gene set enrichment analysis (GSEA) of “TNFR2 non-canonical NF-𝜅B pathway” reactome gene sets, which were the most relevant gene sets identified as enriched in NK cells from the healthy blood donors compared to the COVID-19 patients. False discovery rate (FDR) q-value, normalized enrichment score (NES) and the corresponding nominal *p*-value (NOM *p*-value) are reported (NES = 1.37; NOM *p*-value = 0.001; FDR = 0.231). (**D**) Violin plot representation of the five most differentially expressed receptor genes in NK-T cells from both cohorts of COVID (red) and control (grey) samples. (**E**) Gene set enrichment analysis (GSEA) of “Signaling by G protein-coupled receptor (GPCR)” reactome gene sets, which were the most relevant gene sets identified as enriched in NK-T cells from healthy donors compared to COVID-19 patients. False discovery rate (FDR) q-value, normalized enrichment score (NES) and the corresponding nominal *p*-value (NOM *p*-value) are reported (NES = 1.47; NOM *p*-value = 0.001; FDR = 0.007).

In line with previous reports, we also observed that the composition of NK and NK-T cell subsets differed significantly among COVID-19 patients and healthy donors [20] (Figure 1F). In order to highlight any COVID-19-specific immunophenotypic difference in NK and NK-T cells, we also determined the differentially expressed genes, encoding cell surface proteins, between COVID-19 and control samples (Figure 4A). Particularly, we observed an increased expression of ITGB2, FCGR3, KLRF1, CD247 and CD300A markers and a reduction in CXCR4, CD69, CD48, ICAM1 and S100A10 markers in COVID-derived NK cells with respect to the control (Figure 4B), underlining the heterogeneity of these cell subsets. Of note, the detected transcriptional changes were strictly associated with a reduction in the genes involved in the non-canonical NF-κB pathway mediated by the tumor necrosis factor receptor 2 (TNFR2) (Figure 4C), which modulates the virus-stimulated type I interferon (IFN) production [38] and enforces the NK cell cytotoxicity [39]. We also found an increased expression of CD63, ITGB2, CD37, CD2 and IL23R markers and the reduction in CXCR4, CD69, CD48, ICAM1 and S100A10 markers in COVID-19-derived NK-T cells compared to controls (Figure 4D). Moreover, in the NK-T cells from the heathy blood donors, we observed an enrichment of genes involved in the signaling mediated by the G-protein-coupled receptors (GPCR) (Figure 4E), which control natural killer cell migration in response to infectious or other inflammatory states [40]. All these findings report immunophenotypic changes in T and NK cell subsets, which may reflects variations and dysfunctions in the response to SARS-CoV-2 virus infection.

### 3.4. Monocytes and Their States in COVID-19 Patients

**Figure 5:**
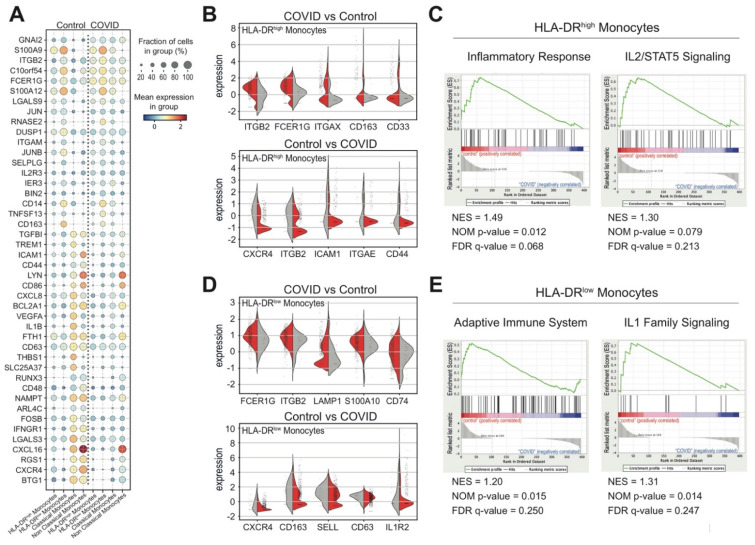
Transcriptional signature of monocyte cell subsets derived from COVID-19 patients at diagnosis. (**A**) Dot plot representation of genes, encoding cell surface proteins and significantly differentially expressed (*p*-value < 0.01) among the indicated monocyte cell subsets from COVID-19 patients and healthy donors. (**B**) Violin plot representation of the five most differentially expressed receptor genes in HLA-DR^high^ monocytes from both cohorts of COVID (red) and control (grey) samples. (**C**) Gene set enrichment analysis (GSEA) of “inflammatory response” and “IL2/STAT5 signaling” reactome gene sets, which were the most relevant gene sets identified as enriched in HLA-DR^high^ monocytes from healthy blood donors compared to COVID-19 patients. False discovery rate (FDR) q-values, normalized enrichment score (NES) and the corresponding nominal *p*-value (NOM *p*-value) are reported within each graph (Inflammatory Response Pathway GeneSet: NES = 1.49; NOM *p*-value = 0.012; FDR = 0.068; IL2/STAT5 Signaling Pathway GeneSet: NES = 1.30; NOM *p*-value = 0.079; FDR = 0.213). (**D**) Violin plot representation of the five most differentially expressed receptor genes in HLA-DR^low^ monocytes cells from both cohorts of COVID (red) and control (grey) samples. (**E**) Gene set enrichment analysis (GSEA) of “adaptive immune system” and “IL1 family signaling” reactome gene sets which resulted to be the most relevant gene sets identified as enriched in HLA-DR^low^ monocytes from healthy blood donors compared to COVID-19 patients. False discovery rate (FDR) q-values, normalized enrichment score (NES) and the corresponding nominal *p*-value (NOM *p*-value) are reported within each graph (Adaptive Immune System Pathway GeneSet: NES = 1.20; NOM *p*-value = 0.015; FDR = IL1 Family Signaling Pathway GeneSet: NES = 1.31; NOM *p*-value = 0.014; FDR = 0.247).

In the last few years, it has been reported that the monocyte compartment is particularly affected in COVID-19 patients [19]. To further investigate the phenotypic alterations of monocytes in response to SARS-CoV-2 virus infection, we divided them into HLA-DR^high^ or HLA-DR^low^ cell types, based on the transcriptional level of the HLA-DRA gene in the studied dataset, and determined the most differentially expressed genes, encoding cell surface proteins, between COVID-19 and control samples (Figure 5A and Figure S10). Specifically, HLA-DR^high^ monocytes, derived from patients in the studied cohort, showed high expression of ITGB2, FCER1G, ITGAX, CD163 and CD44 markers and low transcriptional levels of CXCR4, CD48, ICAM1, ITGAE and CD44 markers compared to healthy blood donors (Figure 5B). Instead, the patient-derived HLA-DR^low^ monocytes were characterized by a high expression of FCER1G, ITGB2, LAMP1, A100A10 and CD74 markers and low levels of CXCR4, CD163, SELL, CD63 and IL2R markers compared to control cells (Figure 5D). Alternative gene signatures were also observed in HLA-DR^high^ and HLA-DR^low^ monocytes in COVID-19 patients. Interestingly, gene set enrichment analysis (GSEA) [41,42] identified an enrichment of “inflammatory response” and “IL2/STAT5 signaling” reactome [43,44] gene sets in HLA-DR^high^ monocytes from healthy donors (Figure 5C), which release proinflammatory cytokines under IL2 stimulation [45]. Additionally, the “adaptive immune system” and “IL1 family signaling” reactome gene sets, which mediate the inflammation in response to several stimuli, including viruses [46], were enriched in HLA-DR^low^ control monocytes (Figure 5E). Taken together, these findings highlight alternative COVID-19-specific activation states of cells that may be associated with disease progression and severity.

## 4. Discussion

Previous studies have reported that SARS-CoV-2 infection generally triggers dysregulated immune responses associated with disease severity [19,20,21,47]. Hence, the identification of predictive immune markers and potential therapeutic targets would contribute to monitoring and preventing the fatal progression of COVID-19. In this study, we performed a targeted RNA sequencing system at the single-cell level (scRNA-Seq) together with a multiparameter flow cytometry analysis to assess alterations in the immune landscape of 50 COVID-19 patients at diagnosis. This integrated experimental approach revealed drastic changes within the main immunological compartments in response to the SARS-CoV-2 infection. Particularly, we observed an expansion of CD8+ and CD4+ T cells, NK cells, HLA-DR^high^ and HLA-DR^low^ monocytes in COVID-19 patients at diagnosis compared to post-COVID and healthy blood donors. Additionally, supervised regression analyses of all demographic, clinical and immunological data, available for our cohort of COVID-19 patients, highlighted features such as age, low level of total and plasma B cells and an increase in HLA-DR^high^ and HLA-DR^low^ monocytes which may have caused a poor clinical outcome. Interestingly, dysfunctional HLA-DR^high^ and HLA-DR^low^ CD14+ monocytes in severe COVID-19 were already characterized using an integrated approach involving mass cytometry (CyTOF), scRNA-Seq and flow cytometry assays [19].

Furthermore, an increase in NK-T cells characterized the former COVID-19 participants after hospitalization in the absence of SARS-CoV-2 virus, as detected by molecular testing, suggesting that these cell subsets might enforce the response against the SARS-CoV-2 infection. Interestingly, we have recently reported that the level of total NK-T cells in peripheral blood significantly correlates with the anti-SARS-CoV-2 IgG level in response to the BNT162b2 (Pfizer–BioNTech) vaccine [48], suggesting that these cell subsets might enhance the antibody production and B cell response, as reported previously [49,50]. Nonetheless, due to relative proportions of PBMCs in the considered assays, it remains uncertain whether the reduced proportion of a population is the result of a decrease in a specific cell type or rather of an increase in another leukocyte subset.

In addition to the expected decrease in total and plasma B cells, we also determined the antigen-specific immune profile against SARS-CoV-2 proteins and identified nodes of interactions, suggesting that the viral proteins do not equally trigger the clonal B cells expansion, but the immune B cell response is mainly developed against specific viral particles. Notably, the B-cell-mediated immunity can be differentially directed towards specific clusters of viral proteins that preferentially expand clones of B cell populations in each COVID-19 patient.

In this work, we also aimed to identify novel COVID-19 specific immune markers for the optimization of monitoring the disease progression and severity. In each main immunological compartment, we identified receptors associated with transcriptional programs of inflammatory response and signaling, mainly repressed or dysfunctional in patient-derived cells. Surprisingly we found that the expression of chemokine receptor CXCR4, which promotes hematopoiesis, cell migration/homing and bone marrow retention [51], was reduced in different immune subsets from COVID-19 patients of our considered cohort. Of interest, an altered expression of various receptors involved in migration, adhesion and activation, including CXCR4, CD63 and CD69, have been reported in COVID-19, highlighting specific immunophenotypes with predictive potential for clinical outcomes [52,53]. Furthermore, in contrast with an increase in both IFN-α and IFN-γ plasma levels, detected in the peripheral blood of COVID-19 patients at the onset of infection, a reduction in the expression of Interferon gamma receptor 1 (IFNGR1) in COVID-19-derived B cells and CD8+ naïve T cells was also detected, suggesting that the sensitivity of immune cells to inflammatory signal molecules might be lower during the infection and that an interferon-based treatment [54] may not be effective for all patients with mild to severe COVID-19. Overall, our data support the idea that there are changes in the immunological landscape between COVID-19 patients at diagnosis and after hospitalization also in relation to healthy uninfected individuals. Moreover, differences in the immune response also point towards different COVID-19-specific immuno markers that correlate with disease severity and might be employed for the optimization of flow cytometry assays for the diagnosis and monitoring of COVID-19 progression. Future studies could elucidate the underlying causes of these different responses that could arise from a history of previous similar infections, or genetic, individual and environmental differences.

## Data Availability

The data presented in this study are available on request from the corresponding author.

## Supplementary Materials

The following are available online at https://www.mdpi.com/article/10.3390/biomedicines9121794/s1. List of Members of the CSS COVID-19 Group, Figures S1–S10 and Tables S1–S3.

## References

[1] Hu B., Guo H., Zhou P., Shi Z.-L. (2021). Characteristics of SARS-CoV-2 and COVID-19. Nat. Rev. Microbiol..

[2] Lee I.T., Nakayama T., Wu C.-T., Goltsev Y., Jiang S., Gall P.A., Liao C.-K., Shih L.-C., Schürch C.M., McIlwain D.R. (2020). ACE2 localizes to the respiratory cilia and is not increased by ACE inhibitors or ARBs. Nat. Commun..

[3] Ou X., Liu Y., Lei X., Li P., Mi D., Ren L., Guo L., Guo R., Chen T., Hu J. (2020). Characterization of spike glycoprotein of SARS-CoV-2 on virus entry and its immune cross-reactivity with SARS-CoV. Nat. Commun..

[4] Shereen M.A., Khan S., Kazmi A., Bashir N., Siddique R. (2020). COVID-19 infection: Emergence, transmission, and characteristics of human coronaviruses. J. Adv. Res..

[5] Yap J.K.Y., Moriyama M., Iwasaki A. (2020). Inflammasomes and Pyroptosis as Therapeutic Targets for COVID-19. J. Immunol..

[6] Aleem A., Slenker A.K. (2021). Monoclonal Antibody Therapy for High-Risk Coronavirus (COVID 19) Patients with Mild to Moderate Disease Presentations.

[7] Ricci D., Etna M., Rizzo F., Sandini S., Severa M., Coccia E. (2021). Innate Immune Response to SARS-CoV-2 Infection: From Cells to Soluble Mediators. Int. J. Mol. Sci..

[8] Huang C., Wang Y., Li X., Ren L., Zhao J., Hu Y., Zhang L., Fan G., Xu J., Gu X. (2020). Clinical features of patients infected with 2019 novel coronavirus in Wuhan, China. Lancet.

[9] Sette A., Crotty S. (2021). Adaptive immunity to SARS-CoV-2 and COVID-19. Cell.

[10] Pan Y., Jiang X., Yang L., Chen L., Zeng X., Liu G., Tang Y., Qian C., Wang X., Cheng F. (2021). SARS-CoV-2-specific immune response in COVID-19 convalescent individuals. Signal Transduct. Target. Ther..

[11] Tay M.Z., Poh C.M., Rénia L., Macary P.A., Ng L.F.P. (2020). The trinity of COVID-19: Immunity, inflammation and intervention. Nat. Rev. Immunol..

[12] Wu Z., McGoogan J.M. (2020). Characteristics of and Important Lessons From the Coronavirus Disease 2019 (COVID-19) Outbreak in China: Summary of a Report of 72 314 Cases From the Chinese Center for Disease Control and Prevention. JAMA.

[13] Shibabaw T., Molla M.D., Teferi B., Ayelign B. (2020). Role of IFN and Complements System: Innate Immunity in SARS-CoV-2. J. Inflamm. Res..

[14] Kaklamanos A., Belogiannis K., Skendros P., Gorgoulis V.G., Vlachoyiannopoulos P.G., Tzioufas A.G. (2021). COVID-19 Immunobiology: Lessons Learned, New Questions Arise. Front. Immunol..

[15] Xiao F., Tang M., Zheng X., Liu Y., Li X., Shan H. (2020). Evidence for Gastrointestinal Infection of SARS-CoV-2. Gastroenterology.

[16] Helms J., Tacquard C., Severac F., Leonard-Lorant I., Ohana M., Delabranche X., Merdji H., Clere-Jehl R., Schenck M., Gandet F.F. (2020). High risk of thrombosis in patients with severe SARS-CoV-2 infection: A multicenter prospective cohort study. Intensive Care Med..

[17] Von Weyhern C.H., Kaufmann I., Neff F., Kremer M. (2020). Early evidence of pronounced brain involvement in fatal COVID-19 outcomes. Lancet.

[18] Xu Z., Shi L., Wang Y., Zhang J., Huang L., Zhang C., Liu S., Zhao P., Liu H., Zhu L. (2020). Pathological findings of COVID-19 associated with acute respiratory distress syndrome. Lancet Respir. Med..

[19] Schulte-Schrepping J., Reusch N., Paclik D., Baßler K., Schlickeiser S., Zhang B., Krämer B., Krammer T., Brumhard S., Bonaguro L. (2020). Severe COVID-19 Is Marked by a Dysregulated Myeloid Cell Compartment. Cell.

[20] Wen W., Su W., Tang H., Le W., Zhang X., Zheng Y., Liu X., Xie L., Li J., Ye J. (2020). Immune cell profiling of COVID-19 patients in the recovery stage by single-cell sequencing. Cell Discov..

[21] Wilk A.J., Rustagi A., Zhao N.Q., Roque J., Martínez-Colón G.J., McKechnie J.L., Ivison G.T., Ranganath T., Vergara R., Hollis T. (2020). A single-cell atlas of the peripheral immune response in patients with severe COVID-19. Nat. Med..

[22] Marshall J.C., Murthy S., Diaz J., Adhikari N.K., Angus D.C., Arabi Y.M., Baillie K., Bauer M., Berry S., Blackwood B. (2020). A minimal common outcome measure set for COVID-19 clinical research. Lancet Infect. Dis..

[23] Levine J.H., Simonds E.F., Bendall S.C., Davis K.L., Amir E.-A.D., Tadmor M.D., Litvin O., Fienberg H.G., Jager A., Zunder E.R. (2015). Data-Driven Phenotypic Dissection of AML Reveals Progenitor-like Cells that Correlate with Prognosis. Cell.

[24] Wolf F.A., Angerer P., Theis F.J. (2018). SCANPY: Large-scale single-cell gene expression data analysis. Genome Biol..

[25] Slovin S., Carissimo A., Panariello F., Grimaldi A., Bouché V., Gambardella G., Cacchiarelli D. (2021). Single-Cell RNA Sequencing Analysis: A Step-by-Step Overview. Springer Protocols Handbooks.

[26] Haghverdi L., Lun A.T.L., Morgan M.D., Marioni J.C. (2018). Batch effects in single-cell RNA-sequencing data are corrected by matching mutual nearest neighbors. Nat. Biotechnol..

[27] Giambra V., Gusscott S., Gracias D., Song R., Lam S.H., Panelli P., Tyshchenko K., Jenkins C.E., Hoofd C., Lorzadeh A. (2018). Epigenetic Restoration of Fetal-like IGF1 Signaling Inhibits Leukemia Stem Cell Activity. Cell Stem Cell.

[28] Van der Maaten L., Hinton G. (2008). Visualizing data using t-SNE. J. Mach. Learn. Res..

[29] Xia X., Wen M., Zhan S., He J., Chen W. (2020). An increased neutrophil/lymphocyte ratio is an early warning signal of severe COVID-19. J. South. Med. Univ..

[30] Chen R., Lan Z., Ye J., Pang L., Liu Y., Wu W., Qin X., Guo Y., Zhang P. (2021). Cytokine Storm: The Primary Determinant for the Pathophysiological Evolution of COVID-19 Deterioration. Front. Immunol..

[31] Rabaan A., Al-Ahmed S., Muhammad J., Khan A., Sule A., Tirupathi R., Mutair A., Alhumaid S., Al-Omari A., Dhawan M. (2021). Role of Inflammatory Cytokines in COVID-19 Patients: A Review on Molecular Mechanisms, Immune Functions, Immunopathology and Immunomodulatory Drugs to Counter Cytokine Storm. Vaccines.

[32] Franzén O., Gan L.-M., Björkegren J.L.M. (2019). PanglaoDB: A web server for exploration of mouse and human single-cell RNA sequencing data. Database.

[33] Lederer K., Castaño D., Atria D.G., Oguin T.H., Wang S., Manzoni T.B., Muramatsu H., Hogan M.J., Amanat F., Cherubin P. (2020). SARS-CoV-2 mRNA Vaccines Foster Potent Antigen-Specific Germinal Center Responses Associated with Neutralizing Antibody Generation. Immunity.

[34] Rajamanickam A., Kumar N.P., Nancy P.A., Selvaraj N., Munisankar S., Renji R.M., Vijayalakshmi V., Murhekar M., Thangaraj J.W.V., Kumar M.S. (2021). Recovery of Memory B-cell Subsets and Persistence of Antibodies in Convalescent COVID-19 Patients. Am. J. Trop. Med. Hyg..

[35] Liu B., Han J., Cheng X., Yu L., Zhang L., Wang W., Ni L., Wei C., Huang Y., Cheng Z. (2020). Reduced numbers of T cells and B cells correlates with persistent SARS-CoV-2 presence in non-severe COVID-19 patients. Sci. Rep..

[36] Zhao J., Zhao J., Mangalam A.K., Channappanavar R., Fett C., Meyerholz D.K., Agnihothram S., Baric R.S., David C.S., Perlman S. (2016). Airway Memory CD4 + T Cells Mediate Protective Immunity against Emerging Respiratory Coronaviruses. Immunity.

[37] Shaim H., Estrov Z., Harris D., Sanabria M.H., Liu Z., Ruvolo P., Thompson P.A., Ferrajoli A., Daher M., Burger J. (2018). The CXCR4–STAT3–IL-10 Pathway Controls the Immunoregulatory Function of Chronic Lymphocytic Leukemia and Is Modulated by Lenalidomide. Front. Immunol..

[38] Jin J., Hu H., Li H.S., Yu J., Xiao Y., Brittain G.C., Zou Q., Cheng X., Mallette F.A., Watowich S.S. (2014). Noncanonical NF-κB Pathway Controls the Production of Type I Interferons in Antiviral Innate Immunity. Immunity.

[39] Almishri W., Santodomingo-Garzon T., Le T., Stack D., Mody C.H., Swain M.G. (2016). TNFα Augments Cytokine-Induced NK Cell IFNγ Production through TNFR2. J. Innate Immun..

[40] Walzer T., Vivier E. (2011). G-protein-coupled receptors in control of natural killer cell migration. Trends Immunol..

[41] Takashima Y., Hamano M., Fukai J., Iwadate Y., Kajiwara K., Kobayashi T., Hondoh H., Yamanaka R. (2020). GSEA-assisted gene signatures valid for combinations of prognostic markers in PCNSL. Sci. Rep..

[42] Damian D., Gorfine M. (2004). Statistical concerns about the GSEA procedure. Nat. Genet..

[43] Good B.M., Van Auken K., Hill D.P., Mi H., Carbon S., Balhoff J.P., Albou L.-P., Thomas P.D., Mungall C.J., A Blake J. (2021). Reactome and the Gene Ontology: Digital convergence of data resources. Bioinformatics.

[44] Joshi-Tope G. (2004). Reactome: A knowledgebase of biological pathways. Nucleic Acids Res..

[45] Espinoza-Delgado I., Bosco M.C., Musso T., Gusella G.L., Longo D.L., Varesio L. (1995). Interleukin-2 and human monocyte activation. J. Leukoc. Biol..

[46] Fields J., Günther S., Sundberg E.J. (2019). Structural Basis of IL-1 Family Cytokine Signaling. Front. Immunol..

[47] Giamarellos-Bourboulis E.J., Netea M.G., Rovina N., Akinosoglou K., Antoniadou A., Antonakos N., Damoraki G., Gkavogianni T., Adami M.-E., Katsaounou P. (2020). Complex Immune Dysregulation in COVID-19 Patients with Severe Respiratory Failure. Cell Host Microbe.

[48] Colucci M., De Santis E., Totti B., Miroballo M., Tamiro F., Rossi G., Piepoli A., De Vincentis G., Greco A., Mangia A. (2021). Associations between Allelic Variants of the Human IgH 3′ Regulatory Region 1 and the Immune Response to BNT162b2 mRNA Vaccine. Vaccines.

[49] Leadbetter E.A., Brigl M., Illarionov P., Cohen N., Luteran M.C., Pillai S., Besra G.S., Brenner M.B. (2008). NK T cells provide lipid antigen-specific cognate help for B cells. Proc. Natl. Acad. Sci. USA.

[50] Miyasaka T., Aoyagi T., Uchiyama B., Oishi K., Nakayama T., Kinjo Y., Miyazaki Y., Kunishima H., Hirakata Y., Kaku M. (2012). A possible relationship of natural killer T cells with humoral immune response to 23-valent pneumococcal polysaccharide vaccine in clinical settings. Vaccine.

[51] Bianchi M.E., Mezzapelle R. (2020). The Chemokine Receptor CXCR4 in Cell Proliferation and Tissue Regeneration. Front. Immunol..

[52] Lourda M., Dzidic M., Hertwig L., Bergsten H., Medina L.M.P., Sinha I., Kvedaraite E., Chen P., Muvva J.R., Gorin J.-B. (2021). High-dimensional profiling reveals phenotypic heterogeneity and disease-specific alterations of granulocytes in COVID-19. Proc. Natl. Acad. Sci. USA.

[53] Silvin A., Chapuis N., Dunsmore G., Goubet A.-G., Dubuisson A., Derosa L., Almire C., Hénon C., Kosmider O., Droin N. (2020). Elevated Calprotectin and Abnormal Myeloid Cell Subsets Discriminate Severe from Mild COVID-19. Cell.

[54] Sodeifian F., Nikfarjam M., Kian N., Mohamed K., Rezaei N. (2021). The role of type I interferon in the treatment of COVID-19. J. Med. Virol..

