# Supplementary material for: COVID-19 Specific Immune Markers Revealed by Single Cell Phenotypic Profiling
Source: Biomedicines. 2021 Nov 29;9(12):1794. doi: 10.3390/biomedicines9121794 (PMC8698462; doi:10.3390/biomedicines9121794)
Supplement: Supplementary file 1 [file biomedicines-09-01794-s001.zip › biomedicines-1432861-supplementary.pdf]

COVID-19 specific immune markers revealed by single cell phenotypic profiling

Francesca Sansico, Mattia Miroballo, Daniele Salvatore Bianco, Francesco Tamiro, Mattia Colucci, Elisabetta De Santis, Giovanni Rossi, Jessica Rosati, Lazzaro Di Mauro, Giuseppe Miscio, Tommaso Mazza, Angelo Luigi Vescovi, Gianluigi Mazzoccoli, Vincenzo Giambra & CSS-COVID 19 Group.

Supplementary Information

including

Members of the CSS COVID-19 Group

Supplementary Figures S1-S10

Supplementary Tables S1-S3

Members of the CSS COVID-19 Group

Paolo E. Alboini, Annibale Antonioni, Filippo Aucella, Giovanni Battista Bochicchio, Cristiano Carbonelli, Massimo Carella, Marco Castori, Antonella Centonze, Elena Chinni, Gianluca Ciliberti, Massimiliano Copetti, Michele Corritore, Salvatore De Cosmo, Leonardo D'Aloiso, Maria M. D'Errico, Angela de Matthaeis, Alfredo Del Gaudio, Annabella Di Giorgio, Vincenzo Giambra, Elvira Grandone, Antonio Greco, Lucia Florio, Andrea Fontana, Vincenzo Inchingolo, Michele Inglese, Maria Labonia, Antonella La Marca, Tiziana Latiano, Maurizio Leone, Evaristo Maiello, Alessandra Mangia, Carmen Marciano, Valentina Massa, Simonetta Massafra, Antonio Mirijello, Grazia Orciuli, Nicola Palladino, Rita Perna, Pamela Piscitelli, Matteo Piemontese, Michele A. Prencipe, Pamela Raggi, Maria Grazia Rodriquenz, Raffaele Russo, Daniele Sancarlo, Annalisa Simeone, Vincenzo Trischitta, Michele Zarrelli, Pasquale Vaira, Doriana Vergara, Angelo Vescovi.

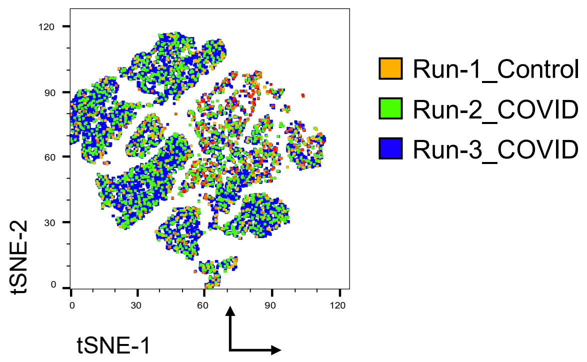

Supplementary Figure S1. tSNE visualization based on the inter-run data.
tSNE plots as in Figure 1A with cells colored according to the sequencing of three independent DNA libraries. These were generated from two cell pools, each derived from 25 different COVID-19 patients as well as a cell pool derived from 27 healthy blood donors as control. Batch effects across different datasets were correct by mutual nearest neighbors (MNN) algorithm through R-based default functions.

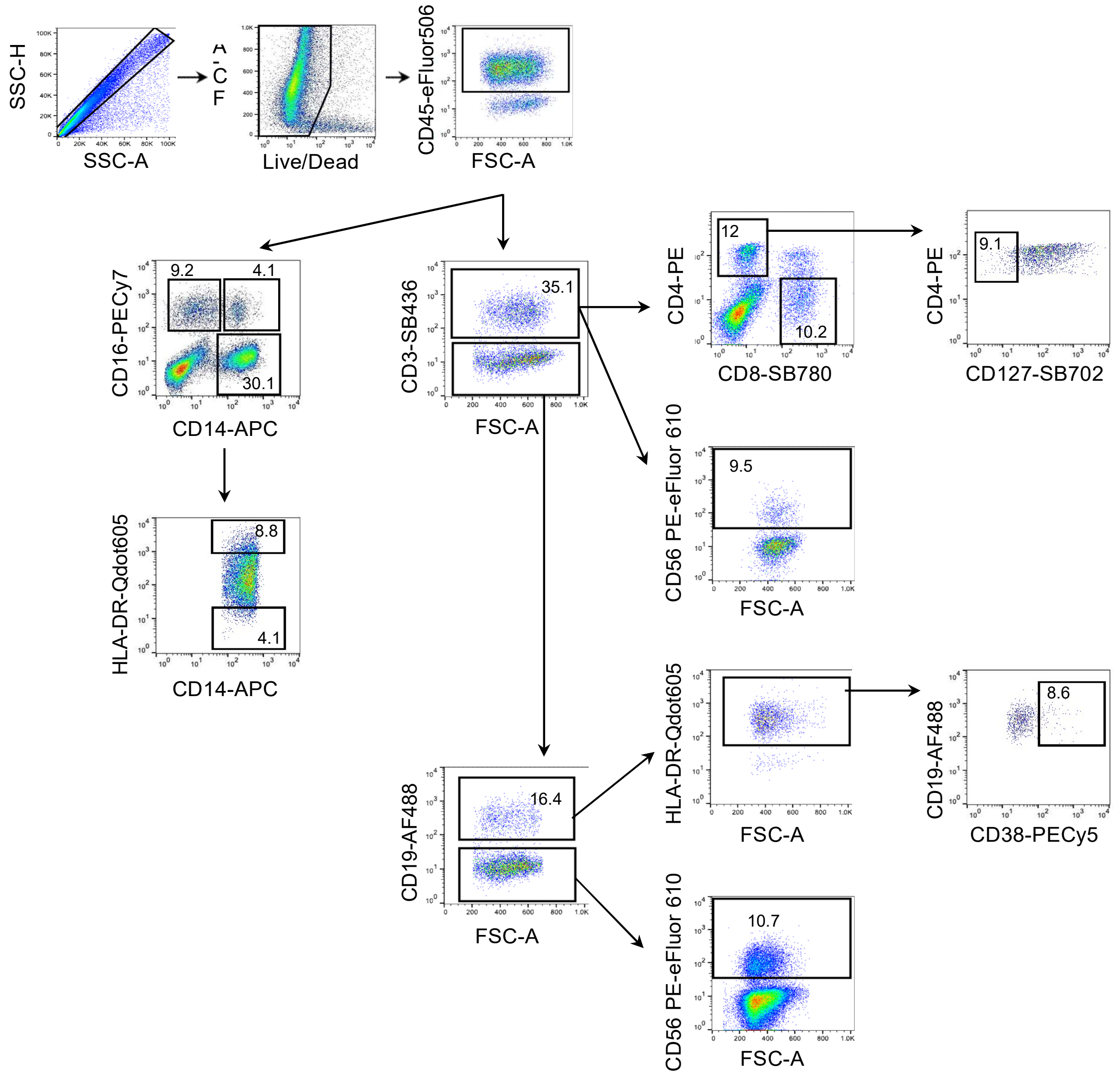

Supplementary Figure S2. Overview of gating strategy for identifying the different subsets in peripheral blood mononuclear cells (PBMCs) with the reported panel (Table S2).
Fluorescence minus one (FMO) controls were used to set up all gates. Singlets were initially discriminated on SSC-H and SSC-A, followed by the exclusion of non-viable cells with Live/Dead far-red fluorescent DNA dye and the identification of CD45+ cell fraction. Subsequent gating identified CD3 + cells, followed by the identification of CD4+CD8- helper T cells and CD4-CD8+ cytotoxic T cells. Within CD4+ cell fraction, regulatory T cells were discriminated as CD127- cells. Natural killer T-cells were also identified as CD56+ cells in the CD3+ fraction. NK cells were recognized as CD3-CD56+CD19-. B cells were identified as CD3-CD19+ HLA-DR+, and plasma B cells were differentiated as CD38^{high} within the subset of B cells. Finally, monocytes were discriminated as classical (CD14+CD16-), intermediate (CD14+CD16+) and non-classical (CD14^{dim}CD16+). Classical Monocytes were subsequently subdivided in HLA-DR^{high} and HLA-DR^{low} cell fractions.

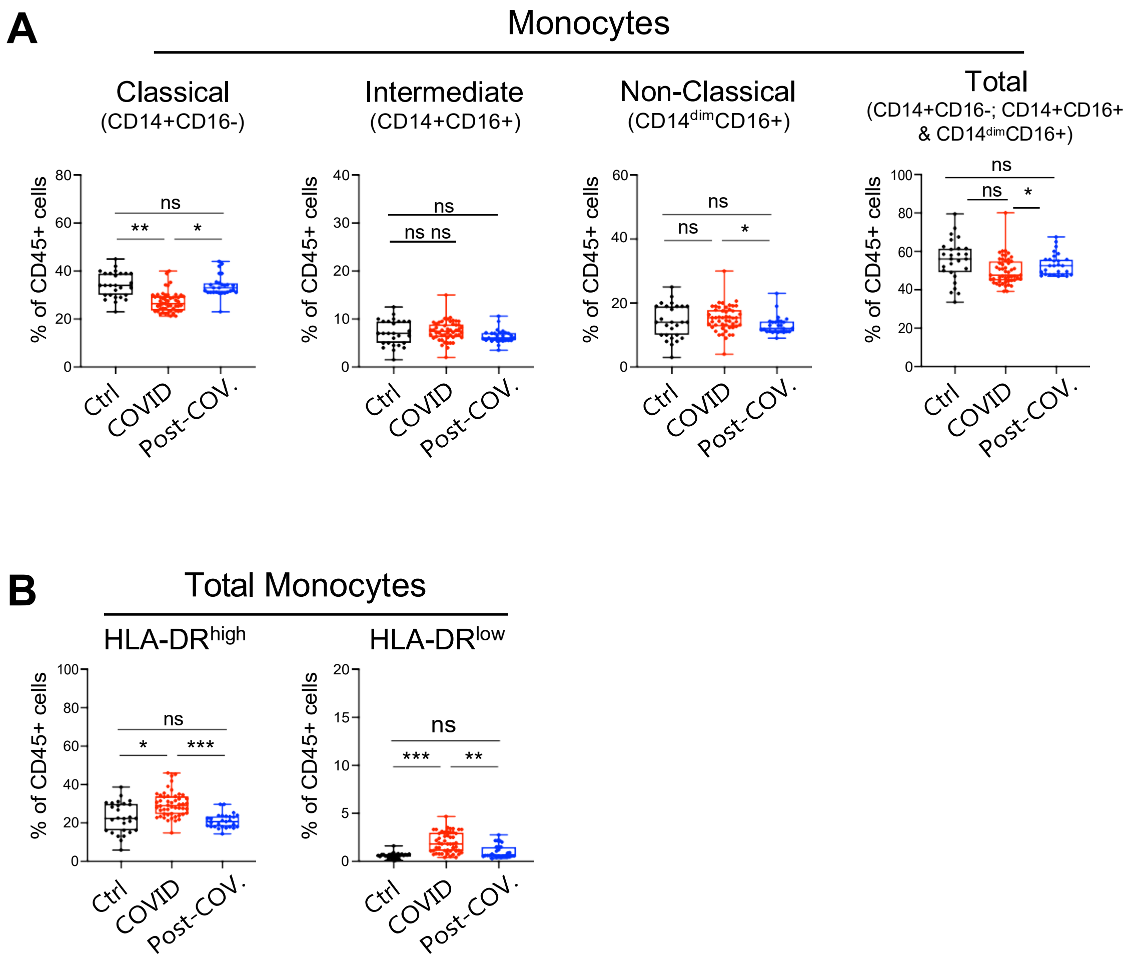

Supplementary Figure S3. Flow cytometry assessment of major lineages and total monocytes in PBMCs from COVID-19 patients and control subjects.
(A) Distribution of total, classical, intermediate and non-classical monocytes in peripheral blood from COVID-19 patients at the diagnosis ("COVID", N=50) and after the first negative molecular test to SARS-CoV-2 ("Post-COV", N=27) and healthy blood donors ("Ctrl", N=27) by multiparameter flow cytometry. Classical monocytes were identified as CD45+CD14+CD16-, intermediate as CD45+CD14+CD16+, and non-classical as CD45+CD14^{dim}CD16+. Instead, total monocytes comprised all three major monocytic lineages. (**B**) Relative proportion of HLA-DR^{high} and HLA-DR^{low} cells within the total monocytic compartment, including classical, intermediate and non-classical monocytes. The Welch's t-test statistical analysis was performed on the percentage values of different cell fractions. *ns, not significant; **, p<0.01; ***, p<0.001 (two-tailed unpaired Welch's t-test).*

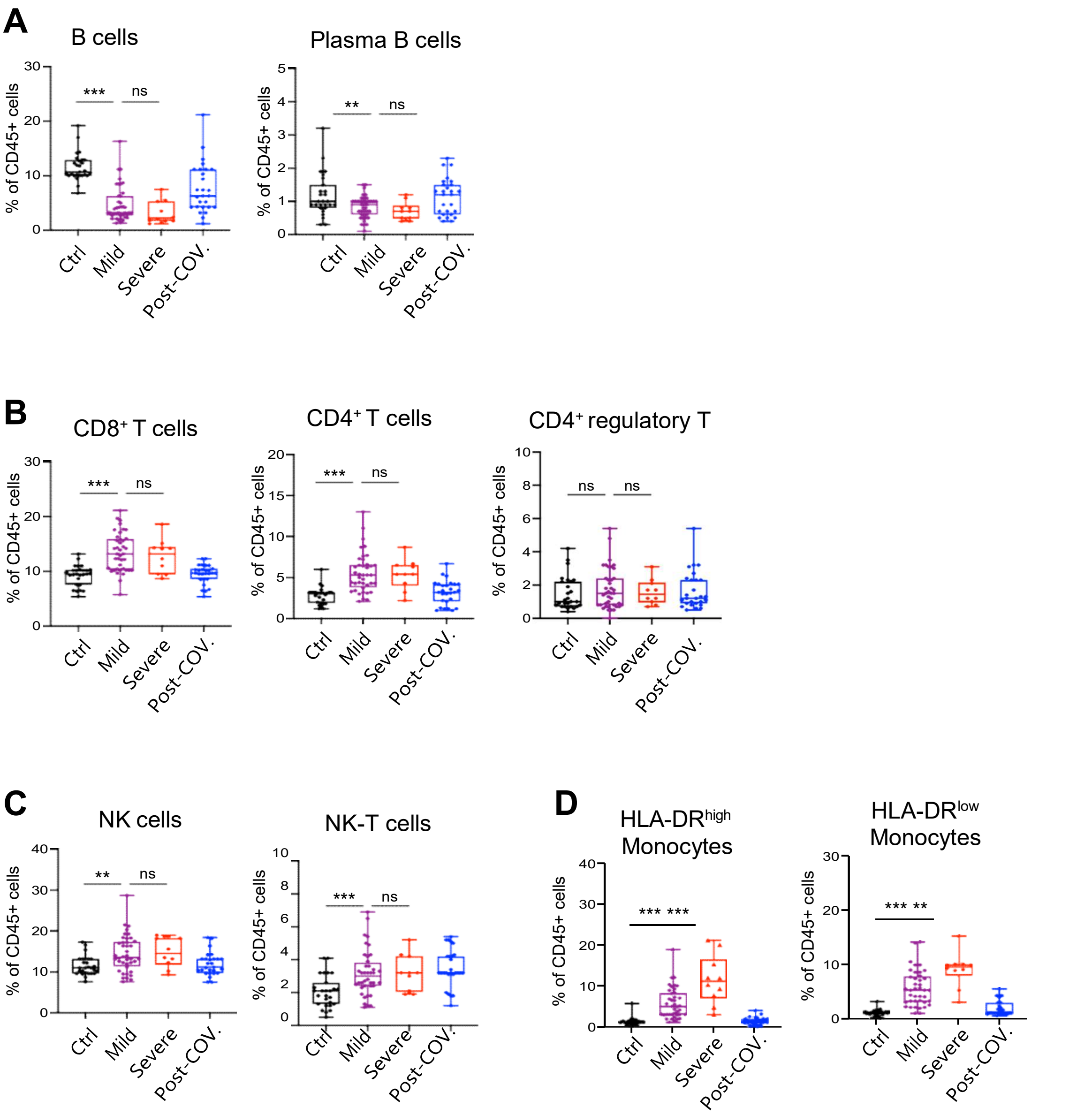

Supplementary Figure S4. Flow cytometry assessment of major cell lineages in PBMCs from COVID-19 patients with mild or severe disease.

Multiparameter flow cytometry analysis of main immune cell populations in peripheral blood from COVID-19 patients grouped according to the disease severity in mild (N=40) and severe (N=10). As controls, healthy donors and former COVID-19 patients (N=27), resulting completely negative to SARS-CoV-2 molecular testing have also been considered and reported as "Ctrl" and "Post-COV" respectively. PBMCs from each individual sample have been labelled after red cell lysis using a panel of 12 fluorophore-conjugated antibodies against lineage-specific cell surface markers in order to identify the B, T, NK and monocytes cell subset. Flow cytometry data are represented in distinct box plots for B-cells and plasma B-cells (**A**); CD8+ T cells, CD4+ T cells and CD4+ regulatory T cells (**B**); NK and NK-T cells (**C**); HLA-DR^{high} Monocytes and HLA-DR^{low} Monocytes (**D**). The Welch's t-test statistical analysis was performed on the percentage values of different cell fractions. *ns, not significant; **, p<0.01; ***, p<0.001 (two-tailed unpaired Welch's t-test).*

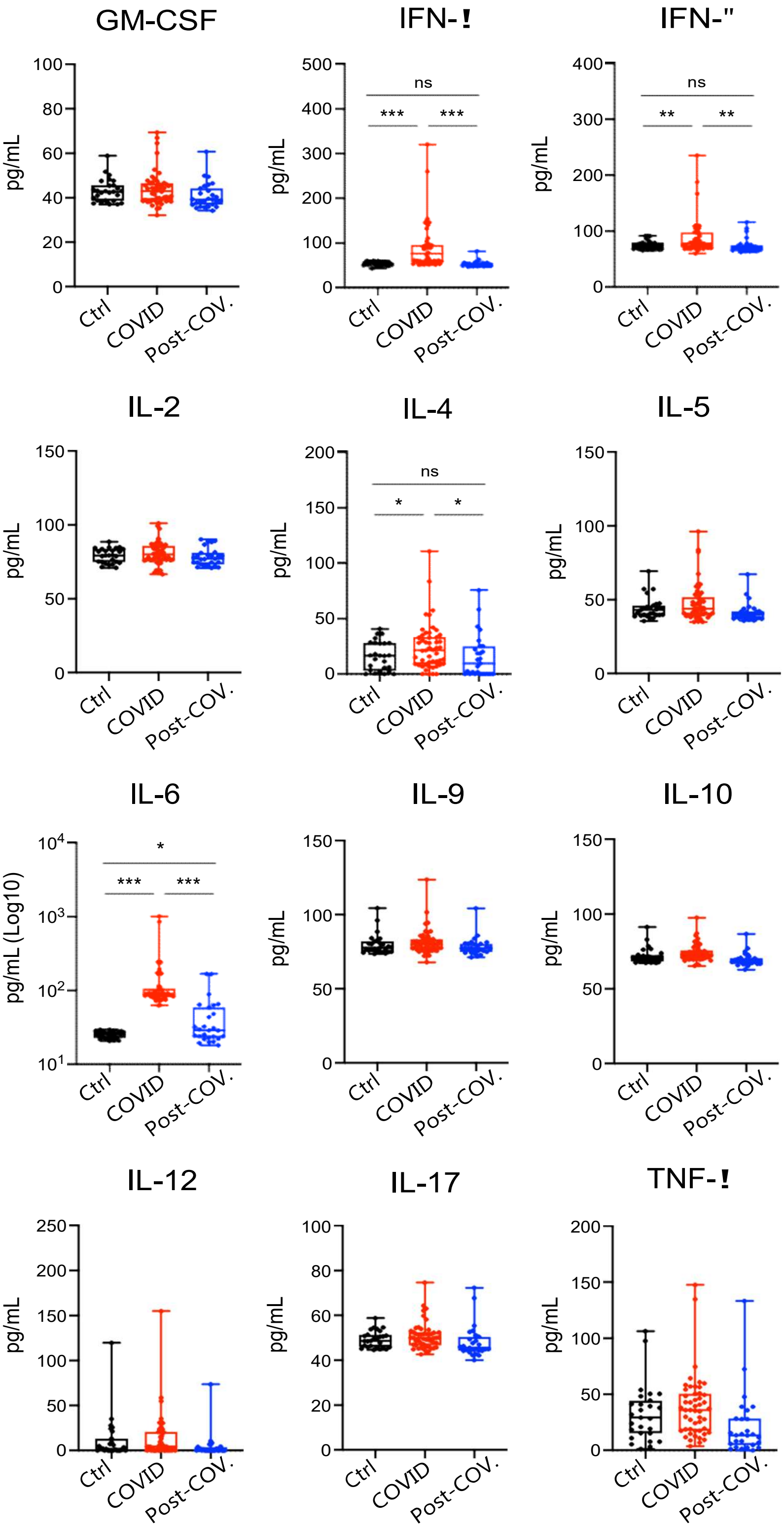

GM-CSF
IFN-!
IFN-"
IL-2
IL-4
IL-5
IL-6
IL-9
IL-10
IL-12
IL-17
TNF-!
pg/mL
pg/mL (Log10)
Ctrl
COVID
Post-COV.
ns
***
**
*

Supplementary Figure S5. Plasma level of representative innate immune cytokines and chemokines in COVID-19 patients and controls

The concentration of soluble human cytokines and chemokines in the plasma of COVID-19 patients (COVID, N=50) at the diagnosis and after resulting negative completely negative to SARS-CoV-2 molecular testing (Post-COV, N=27), as well as healthy donors as control (N=27) was simultaneously determined by flow cytometry analysis based on MACSPlex Capture Beads (Miltenyi Biotec Inc.). Through this assay, GM-CSF, IFN-α, IFN-γ, IL2- IL-4, IL-5, IL-6, IL-9, IL-10, IL-12, IL-17 and TNF-α soluble analytes were detected using a cocktail of various fluorescently labelled bead populations, each coated with specific antibodies. The Welch's t-test statistical analysis was performed on the concentration values of different human cytokines and chemokines. *ns, not significant; *, $p<0.05$; **, $p<0.01$; ***, $p<0.001$ (two-tailed unpaired Welch's t-test).*

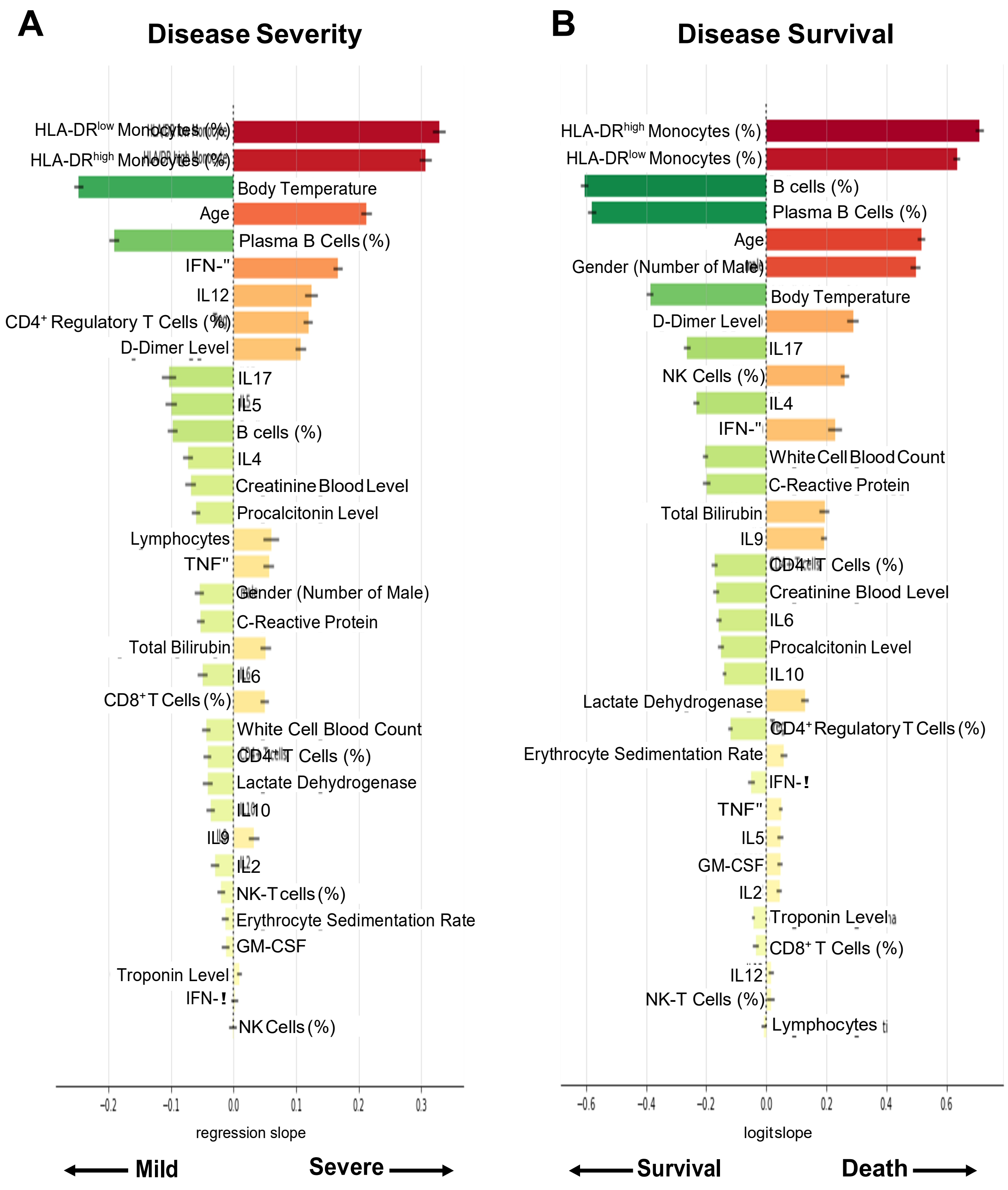

A
Disease Severity
HLA-DRlow Monocytes (%)
HLA-DRhigh Monocytes (%)
Body Temperature
Age
Plasma B Cells (%)
IFN-"
IL12
CD4+ Regulatory T Cells (%)
D-Dimer Level
IL17
IL5
B cells (%)
IL4
Creatinine Blood Level
Procalcitonin Level
Lymphocytes
TNF"
Gender (Number of Male)
C-Reactive Protein
Total Bilirubin
IL6
CD8+ T Cells (%)
White Cell Blood Count
CD4+ T Cells (%)
Lactate Dehydrogenase
IL10
IL9
IL2
NK-T cells (%)
Erythrocyte Sedimentation Rate
GM-CSF
Troponin Level
IFN-!
NK Cells (%)
-0.2
-0.1
0.0
0.1
0.2
0.3
regression slope
Mild
Severe
B
Disease Survival
HLA-DRhigh Monocytes (%)
HLA-DRlow Monocytes (%)
B cells (%)
Plasma B Cells (%)
Age
Gender (Number of Male)
Body Temperature
D-Dimer Level
IL17
NK Cells (%)
IL4
IFN-"
White Cell Blood Count
C-Reactive Protein
Total Bilirubin
IL9
CD4+ T Cells (%)
Creatinine Blood Level
IL6
Procalcitonin Level
IL10
Lactate Dehydrogenase
CD4+ Regulatory T Cells (%)
Erythrocyte Sedimentation Rate
IFN-!
TNF"
IL5
GM-CSF
IL2
Troponin Level
CD8+ T Cells (%)
IL12
NK-T Cells (%)
Lymphocytes
-0.6
-0.4
-0.2
0.0
0.2
0.4
0.6
logit slope
Survival
Death

Supplementary Figure S6. Supervised regression analysis identifies statistically significant correlations between disease severity and clinical landscapes of studied COVID-19 patients. In order to highlight features that may have caused the increased morbidity in the considered COVID-19 patients, we performed a supervised regression using all demographic, clinical and immunological data that were available for our cohort. All parameters were integrated, scaled, and used to train a bootstrap aggregation (Bagging) model to predict the WHO-derived score of each patient, representing the severity of COVID-19 disease. As base learners, 1000 Lasso regressors were trained on different bootstrapped samples and the model performance was tested on both the training set and on an out-of-bag reconstructed test-set (achieving respectively an R^2 score of 0.78 and 0. 23), to be sure that the model is representative of our cohort. Subsequently, Lassos slopes determined the importance of each feature in the regression. In **A**, the bar-plot reports the slopes of all considered feature for each COVID-19 patients related to disease severity. Specifically, the positive slope features in red are associated with the most severe grade of COVID-19 and the negative slope parameters in green highlight more protective features, inversely correlated with the disease severity. The horizontal black lines at the end of the bars are the confidence intervals (α=95%) of the mean slope estimation. The correlations between the highest score of disease and the monocyte levels as well as the patient's age have also been confirmed by the Kendall correlation test (p-value < 0.05). In **B**, the bar-plot reports the slopes of all considered feature for each COVID-19 patients related to disease survival. Specifically, the positive slope features are associated to the patient mortality, while the negative slope features are associated to the patient survival. The associations of mortality with monocyte levels and the patient's age as well as the correlations between patient survival and B-cell levels have also been confirmed by the Mann-Whitney test (p-value < 0.05).

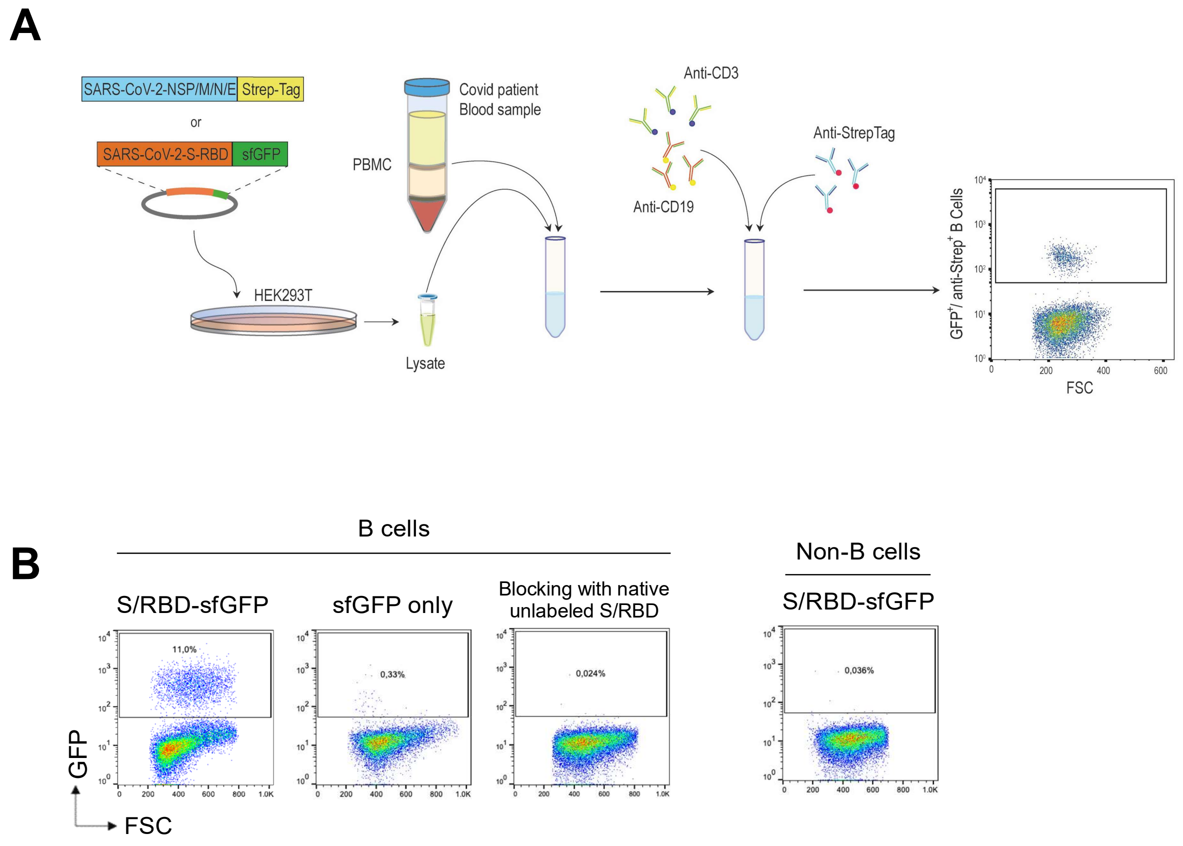

Supplementary Figure S7. Schematic overview of experimental approach for the detection of B cells interacting with SARS-CoV-2 structural and non-structural proteins by flow cytometry.

A) HEK-293T cells were transiently transfected with pcDNA3.1 vectors, encoding the receptor binding domain (RBD) of SARS-CoV-2 protein S (spike) fused with sfGFP fluorescent marker or structural (RBD-S, M, N and E) and non-structural (NSP1, NSP2, NSP4, NSP5, NSP7, NSP8, NSP9, NSP10, NSP11, NSP12, NSP13, NSP14, NSP15) SARS-CoV-2 recombinant proteins with Strep-Tag II synthetic peptide. Afterwards, the recombinant SARS-CoV-2 proteins was purified after 2 days from transfection and employed for the immunostaining of human PBMCs after red blood cell lysis. Initially, cells interacted with the recombinant SARS-CoV-2 proteins or sfGFP/ Strep-Tag II only as control. Subsequently after washing, cells were labelled with a panel of fluorophore-conjugated antibodies against Strep-Tag as well as CD3 and CD19 cell surface markers. **B**) Flow cytometry plots of B cells from a representative sample after interaction with the recombinant sfGFP-tagged SARS-CoV-2 S/RBD protein, only sfGFP as control and following the blocking of binding with native unlabeled SARS-CoV-2 S/RBD protein. Plot of non-B cells from the same sample after interaction with the recombinant sfGFP-tagged SARS-CoV-2 S/RBD protein is also reported. B cells were determined in the CD19+CD3- cell fraction. Boolean gate was applied to identify non-B cells using the FlowJo (Becton Dickinson) software.

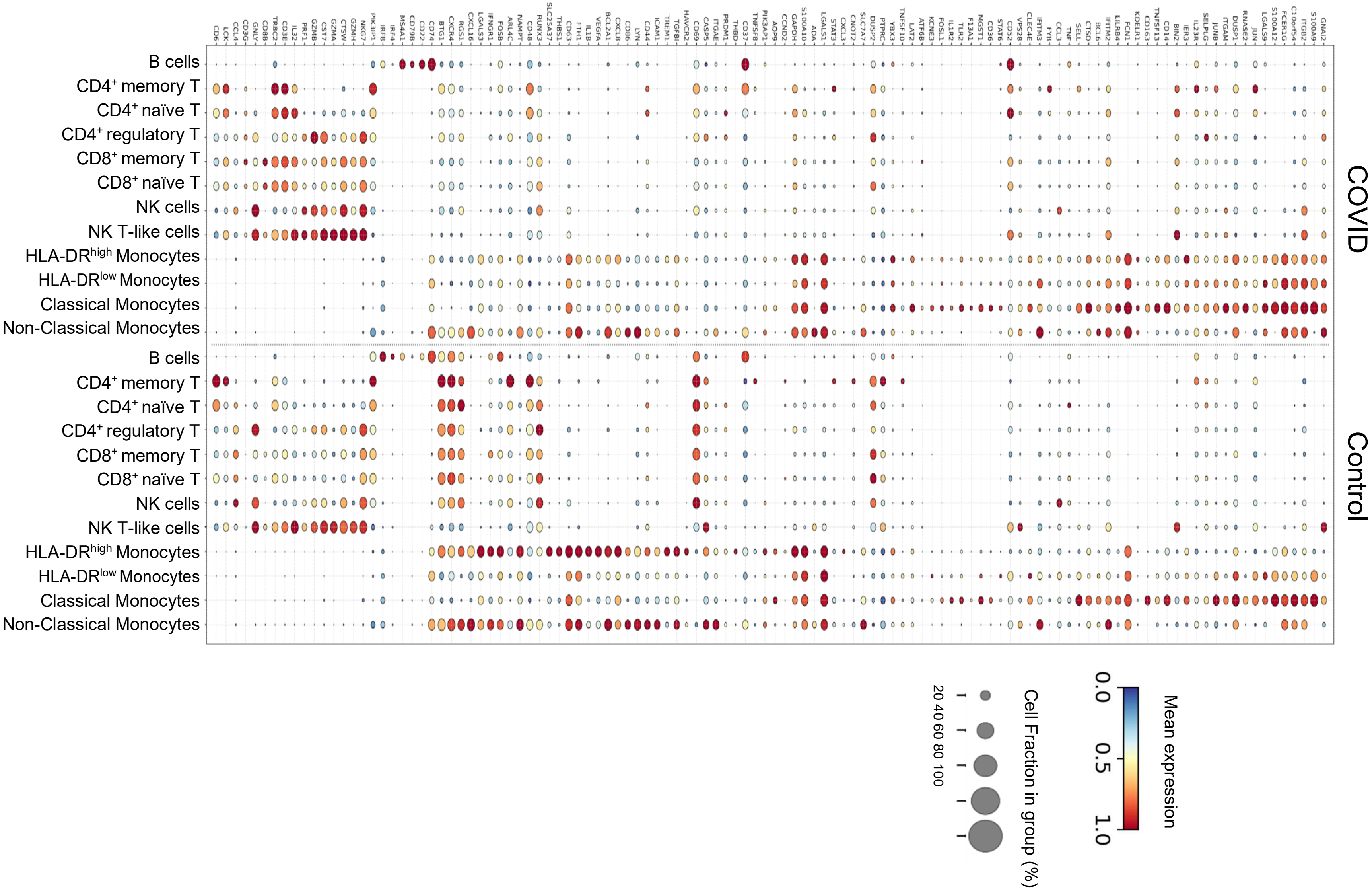
B cells
CD4+ memory T
CD4+ naïve T
CD4+ regulatory T
CD8+ memory T
CD8+ naïve T
NK cells
NK T-like cells
HLA-DRhigh Monocytes
HLA-DRlow Monocytes
Classical Monocytes
Non-Classical Monocytes
COVID
Control
Cell Fraction in group (%)
20 40 60 80 100
Mean expression
0.0 0.5 1.0

Supplementary Figure S8. Dot plot of the main differentially expressed genes for the indicated cell subsets from COVID-19 patients and healthy donors by scRNA-Seq profiling. Dot plots of the main differentially expressed genes of BD Rhapsody^{TM} Immune Response Panel for the indicated PBMC subsets from COVID-19 patients and healthy donors. The colour and the size of each dot represent the normalized average expression levels and the percentage of cells expressing a given gene respectively. On the vertical axis are listed the genes returned as significantly differentially expressed (p value < 0.01) in at least one of the sub-populations, while on the horizontal axis are displayed the cellular populations grouped for COVID or control cohort.

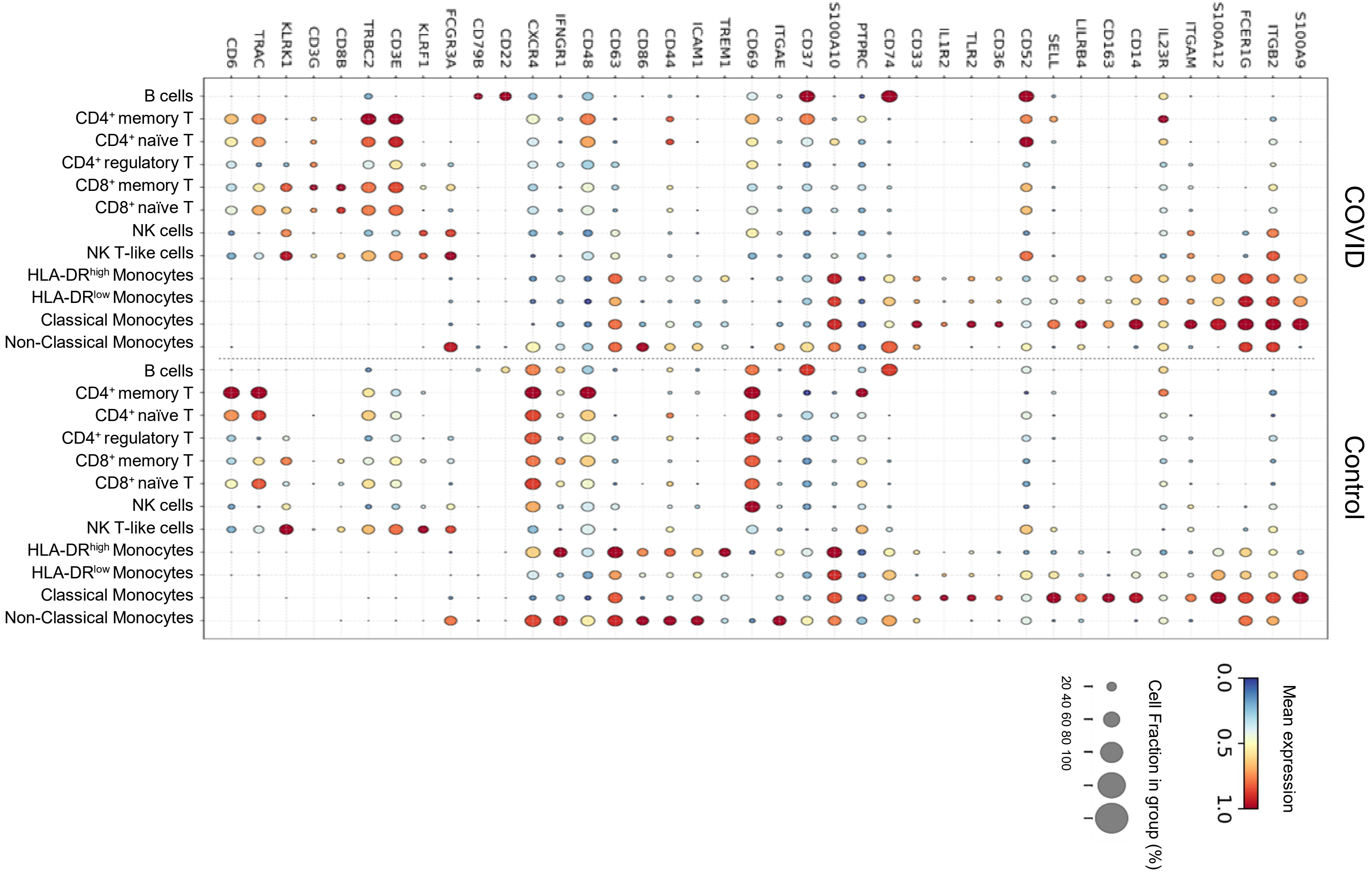

CD6
TRAC
KLRK1
CD3G
CD8B
TRBC2
CD3E
KLRF1
FCGR3A
CD79B
CD22
CXCR4
IFNGR1
CD48
CD63
CD86
CD44
ICAM1
TREM1
CD69
ITGAE
CD37
S100A10
PTPRC
CD74
CD33
IL1R2
TLR2
CD36
CD52
SELL
LILRB4
CD163
CD14
IL23R
ITGAM
S100A12
FCER1G
ITGB2
S100A9
B cells
CD4+ memory T
CD4+ naïve T
CD4+ regulatory T
CD8+ memory T
CD8+ naïve T
NK cells
NK T-like cells
HLA-DRhigh Monocytes
HLA-DRlow Monocytes
Classical Monocytes
Non-Classical Monocytes
COVID
Control
Mean expression
0.0
0.5
1.0
Cell Fraction in group (%)
20 40 60 80 100

Supplementary Figure S9. Dot plot of genes, encoding cell surface proteins and differentially expressed in the indicated cell subsets from COVID-19 patients and healthy donors by scRNA-Seq profiling.

Dot plots of selected genes of BD Rhapsody™ Immune Response Panel, which encodes cell surface proteins and are differentially expressed in the indicated cell subsets of COVID-19 patients and healthy donors. The colour and the size of each dot represent the normalized average expression levels and the percentage of cells expressing a given gene respectively. On the vertical axis are listed the genes returned as significantly differentially expressed (p value < 0.01) in at least one of the sub-populations, while on the horizontal axis are displayed the cellular populations grouped for COVID or control cohort.

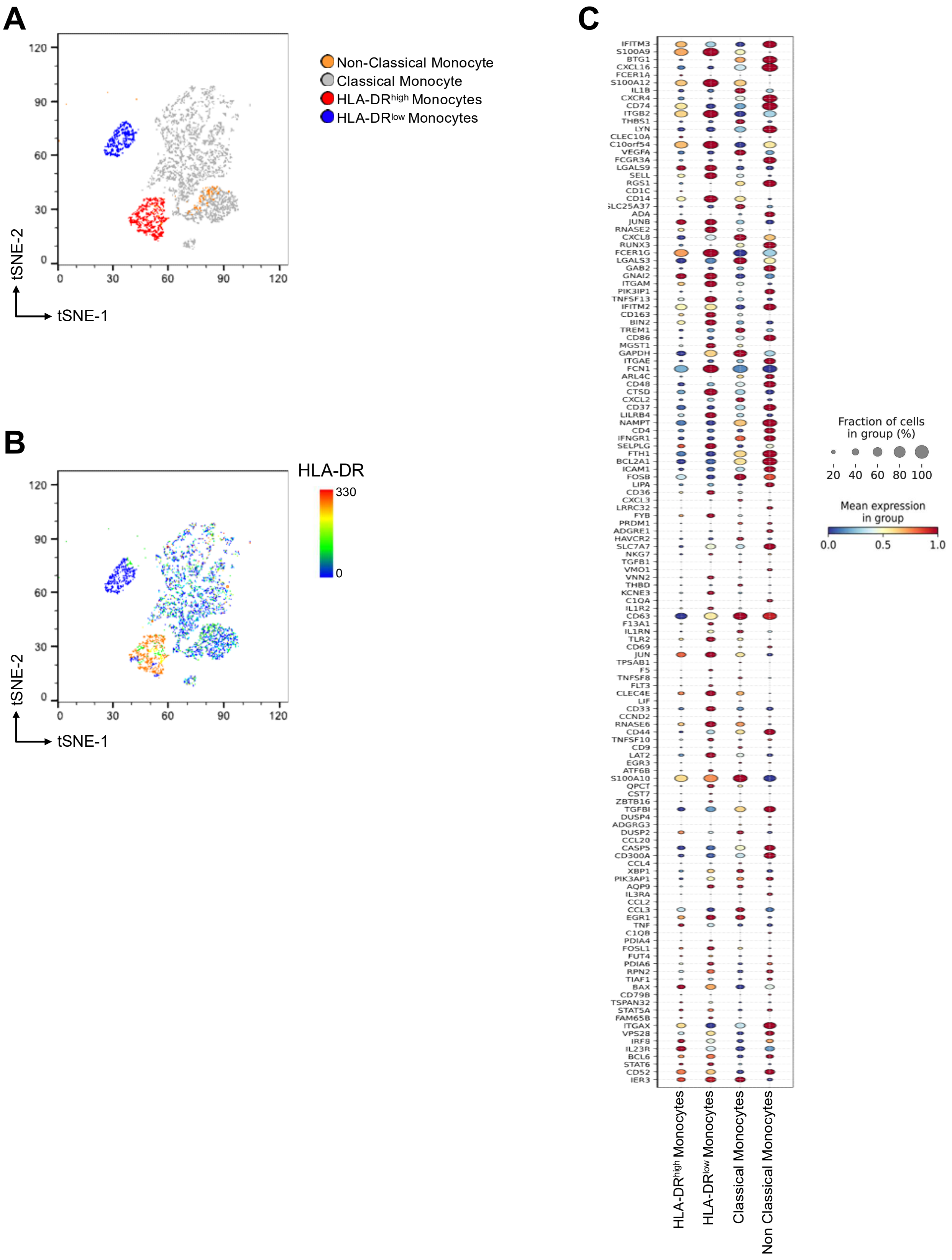
A
120
90
60
30
0
0
30
60
90
120
tSNE-2
tSNE-1
Non-Classical Monocyte
Classical Monocyte
HLA-DRhigh Monocytes
HLA-DRlow Monocytes
B
HLA-DR
330
0
C
IFITM3
S100A9
BTG1
CXCL16
FCER1A
S100A12
IL1B
CXCR4
CD74
ITGB2
THBS1
LYN
CLEC10A
C10orf54
VEGFA
FCGR3A
LGALS9
SELL
RGS1
CD1C
CD14
LC25A37
ADA
JUNB
RNASE2
CXCL8
RUNX3
FCER1G
LGALS3
GAB2
GNAI2
ITGAM
PIK3IP1
TNFSF13
IFITM2
CD163
BIN2
TREM1
CD86
MGST1
GAPDH
ITGAE
FCN1
ARL4C
CD48
CTSD
CXCL2
CD37
LILRB4
NAMPT
CD4
IFNGR1
SELPLG
FTH1
BCL2A1
ICAM1
FOSB
LIPA
CD36
CXCL3
LRRC32
FYB
PRDM1
ADGRE1
HAVCR2
SLC7A7
NKG7
TGFB1
VMO1
VNN2
THBD
KCNE3
C1QA
IL1R2
CD63
F13A1
IL1RN
TLR2
CD69
JUN
TPSAB1
F5
TNFSF8
FLT3
CLEC4E
LIF
CD33
CCND2
RNASE6
CD44
TNFSF10
CD9
LAT2
EGR3
ATF6B
S100A10
QPCT
CST7
ZBTB16
TGFBI
DUSP4
ADGRG3
DUSP2
CCL20
CASP5
CD300A
CCL4
XBP1
PIK3AP1
AQP9
IL3RA
CCL2
CCL3
EGR1
TNF
C1QB
PDIA4
FOSL1
FUT4
PDIA6
RPN2
TIAF1
BAX
CD79B
TSPAN32
STAT5A
FAM65B
ITGAX
VPS28
IRF8
IL23R
BCL6
STAT6
CD52
IER3
HLA-DRhigh Monocytes
HLA-DRlow Monocytes
Classical Monocytes
Non Classical Monocytes
Fraction of cells
in group (%)
20
40
60
80
100
Mean expression
in group
0.0
0.5
1.0

Supplementary Figure S10. Cell distribution and gene expression profiling of monocyte subsets identified by scRNA-Seq assay.

A) tSNE visualization of monocytes identified by the scRNA-Seq profile from two groups of subjects, COVID-19 patients (N=50) and healthy blood donors (N=27) as control. In the graph, each dot represents a single cell, colored according to cell type. Classical and non-classical subsets of monocytes were manually annotated based on the expression of gene markers as references according to the PanglaoDB web server [32]. HLA-DR^{high} and HLA-DR^{low} monocytes were discriminated based on the transcriptional level of HLA-DRA gene. **B**) t-SNE plot of individual monocytes colored according to expression level of HLA-DRA gene. **C**) Dot plot representation of genes of BD Rhapsody^{TM} Immune Response Panel, which are significantly differentially expressed (p value < 0.01) among the indicated monocyte cell subsets.

Disease severity score	MILD 0	MILD 1	SEVERE 2	SEVERE 3	TOT
**DEMOGRAPHICS**					
COVID-19 patients	9 (18%)	31 (62%)	5 (10%)	5 (10%)	**50**
Post COVID-19 in this study	7 (26%)	17 (63%)	3 (11%)	0	**27**
Male	8 (26.6%)	18 (60%)	0	4 (13.3%)	**30**
Female	1 (5%)	13 (65%)	5 (25%)	1 (5%)	**20**
Average Age, Male (years)	55.5	67.8	-	76	
Average Age, Female (years)	39	68.3	64.2	79	
**CLINICAL DATA**					
Body Temperature (°C)	37 ± 0.9	37.3 ± 1.2	37 ± 0.4	36.4 ± 1.4	
White Cell Blood Count (cells/mcL)	6.6 ± 4.3	12.6 ± 18.4	8 ± 4.1	7.1 ± 1.7	
Lymphocytes (cells/mcL)	1.2 ± 0.8	2.8 ± 6.7	2.8 ± 3.2	1.5 ± 0.7	
Total Bilirubin (mg/dL)	0.8 ± 0.6	0.6 ± 0.5	0.6 ± 0.2	0.7 ± 0.3	
Creatinine (mg/dL)	1.1 ± 0.6	1.3 ± 1.5	2 ± 3.1	0.8 ± 0.2	
C-Reactive Protein (mg/L)	4.1 ± 2.3	8.5 ± 9.9	2.5 ± 2.5	7.4 ± 6	
Erythrocyte Sedimentation Rate (mm/h)	52 ± 32.5	37 ± 32.8	23.2 ± 22	40.4 ± 6.7	
D-Dimer (ng/mL)	1500 ± 1852	3940 ± 12237	900 ± 1024	9744 ± 17014	
Procalcitonin (ng/mL)	3.3 ± 9.1	0.4 ± 1	0.5 ± 0.9	0.2 ± 0.1	
Troponin (ng/L)	6.5 ± 12.7	9.6 ± 17.3	6 ± 8.2	15.2 ± 30.4	
Lactate Dehydrogenase (U/L)	260.5 ± 120.8	227.1 ± 194	164.6 ± 94.4	190.4 ± 100	
**COMORBIDITIES**					
Diabetes	0	1 (3,2%)	1 (20%)	0	
Hypertension	1 (11%)	3 (9,6%)	1 (20%)	1 (20%)	
Other	1 (11%)	9 (29%)	2 (40%)	0	
**CYTOKINES CHEMOKINES**					
GM-CSF (pg/mL)	43.9 ± 8.6	44.3 ± 8.5	40.5 ± 2.4	46 ± 4	
IFN-α (pg/mL)	99.2 ± 88	99.6 ± 86.9	182 ± 208	161 ± 215	
IFN-γ (pg/mL)	88.5 ± 16.7	90.8 ± 38.3	77.9 ± 12	84 ± 15.3	
IL2 (pg/mL)	79.1 ± 6	81.5 ± 8.4	77.3 ± 6.2	83.3 ± 3.4	
IL4 (pg/mL)	21.6 ± 14.6	27.5 ± 25.1	14.8 ± 6.6	21.5 ± 7.7	
IL5 (pg/mL)	49.4 ± 13.8	47.7 ± 14	42.6 ± 3.7	50 ± 7.1	
IL6 (pg/mL)	90.6 ± 32.5	162 ± 210	100 ± 39.6	116 ± 73.1	
IL9 (pg/mL)	79.9 ± 2.9	82.1 ± 10.6	77.4 ± 1.6	83.1 ± 3.2	
IL10 (pg/mL)	76.4 ± 11.1	77.2 ± 15.5	72.4 ± 4.4	73.3 ± 1.1	
IL12 (pg/mL)	10.9 ± 12	16 ± 30.2	9.4 ± 14.7	11.7 ± 10.3	
IL17 (pg/mL)	51 ± 4.9	53.2 ± 13.2	48.4 ± 2.5	49.7 ± 2.5	
TNFα (pg/mL)	32.2 ± 14.3	39.6 ± 34.2	31.1 ± 17.3	40 ± 3.7	

Supplementary Table S1. Demographic distribution and clinical features of considered 50 COVID-19 patients at the diagnosis.

Whole blood samples were collected from 50 COVID-19 patients at the onset of infection within 5 days from receiving a positive result to SARS-CoV-2 molecular testing at a large medical center in Southern Italy. The patients were subsequently subdivided by disease severity according to the WHO clinical progression scale [22] in "Mild" (0 = symptomatic without mask; 1= symptomatic with mask) or "Severe" (2= Hospitalization and intensive therapy; 3=dead) depending on the peak of infection. Samples from 27 former COVID-19 patients, resulting completely negative to SARS-CoV-2 molecular testing were also collected and reported as "Post COVID-19". In the Table, the average values and standard deviations of complete blood counts and levels of indicated analytes are reported for all COVID-19 patients subdivided by disease severity at the onset of infection. Finally, blood samples from 27 uninfected healthy donors with the median age of 45 years and 59% of male were included as controls. All participants to this study are Caucasian from the same geographic area in Southern Italy.

Laser	Detector	Fluorophore	Marker	Antibody Clone	Company	Catalog #
405	448/59	Super Bright 436	CD3	SK7	eBioscience™	62-0036-41
	525/50	eFluor 506	CD45	HI30	eBioscience™	69-0459-41
	620/29	Qdot 605	HLA-DR	Tü36	eBioscience™	Q10052
	710/45	Super Bright 702	CD127	eBioRDR5	eBioscience™	67-1278-42
	755 LP	Super Bright 780	CD8	53-6.7	eBioscience™	78-0081-82
488	513/26	Alexa Fluor 488	CD19	SJ25C1	eBioscience™	53-0198-42
	795/70	PE-Cyanine7	CD16	eBioCB16 (CB16)	eBioscience™	25-0168-42
561	579/16	PE	CD4	RPA-T4	eBioscience™	12-0049-42
	614/20	PE-eFluor 610	CD56	CMSSB	eBioscience™	61-0567-42
	692/75	PE-Cyanine5	CD38	HIT2	eBioscience™	15-0389-42
640	671/30	APC	CD14	61D3	eBioscience™	17-0149-41
	722/44	Alexa Fluor 700	CD11b	CBRM1/5	eBioscience™	56-0113-42
	795/70	APC-eFluor 780	Live / Dead		eBioscience™	L34975

Supplementary Table S2. Panel of cell surface markers and fluorophore-conjugated antibodies used in the multiparameter flow cytometry assessment of PBMCs. APC, allophycocyanine; PE, phycoerythrin.

Cell Type	Gating Strategy
B cells	CD45+ CD3- CD19+ HLA-DR+
Plasma B cells	CD45+ CD3- CD19+ HLA-DR+CD38^{high}
NK cells	CD45+ CD3- CD56+ CD19-
NK T-like cells	CD45+ CD3+ CD56+
CD4+ T cells	CD45+ CD3+ CD4+ CD8-
CD4+ Regulatory T cells	CD45+ CD3+ CD4+ CD8- CD127-
CD8+ T cells	CD45+ CD3+ CD8+ CD4-
Classical Monocytes	CD45+ CD14+ CD16-
Intermediate Monocytes	CD45+ CD14+ CD16+
Non-Classical Monocytes	CD45+ CD14^{dim} CD16+
HLA-DR^{high} Classical Monocytes	CD45+ CD14+ CD16- HLA-DR^{high}
HLA-DR^{low} Classical Monocytes	CD45+ CD14+ CD16- HLA-DR^{low}

Supplementary Table S3. Gating strategy for the identification of different cell subpopulations of PBMCs with the reported panel (Table S2).

Fluorescence Minus One (FMO) controls were used to set up all gates. Singlets were initially discriminated on SSC-H and SSC-A, followed by the exclusion of non-viable cells with Live/Dead far-red fluorescent DNA dye. Subsequent gating identifies CD45+ cells, followed by the identification of CD3+CD4+CD8- and CD3+CD4-CD8+ cytotoxic T cells. Within CD4+ T cell fraction, regulatory T cells were discriminated as CD127- cells. Natural killer (NK) T-cells were also identified as CD56+ cells in the CD3+ fraction. NK cells were recognized as CD3-CD56+CD19-. B cells were identified as CD3-CD19+HLA-DR+ and plasma B cells were differentiated as CD38high within B cell subset. Finally, monocytes were discriminated as classical (CD14+CD16-), intermediate (CD14+CD16+) and non-classical (CD14^{dim}CD16+). Classical Monocytes were subsequently subdivided in HLA-DR^{high} and HLA-DR^{low} cell fractions.
